# Effect of phytoplankton size diversity on primary productivity in the North Pacific: trait distributions under environmental variability

**DOI:** 10.1111/ele.13167

**Published:** 2018-10-17

**Authors:** Bingzhang Chen, Sherwood Lan Smith, Kai W. Wirtz

**Affiliations:** ^1^ Research Center for Global Change Research JAMSTEC (Japan Agency for Marine‐Earth Science and Technology) 3173‐25 Showa‐machi, Kanazawa‐ku Yokohama 236‐0001 Japan; ^2^ Helmholtz Centre for Coastal and Materials Research (HZG) Geesthacht Germany

**Keywords:** Biodiversity, ecosystem functioning, evenness, ocean model, phytoplankton size

## Abstract

While most biodiversity and ecosystem functioning (BEF) studies have found positive effects of species richness on productivity, it remain unclear whether similar patterns hold for marine phytoplankton with high local richness. We use the continuous trait‐based modelling approach, which assumes infinite richness and represents diversity in terms of the variance of the size distribution, to investigate the effects of phytoplankton size diversity on productivity in a three‐dimensional ocean circulation model driven by realistic physics forcing. We find a slightly negative effect of size diversity on primary production, which we attribute to several factors including functional trait‐environment interactions, flexible stoichiometry and the saturation of productivity at low diversity levels. The benefits of trait optimisation, whereby narrow size distributions enhance productivity under relatively stable conditions, tend to dominate over those of adaptive capacity, whereby greater diversity enhances the ability of the community to respond to environmental variability.

## Introduction

Studies on the relationships between biodiversity and ecosystem functioning (BEF) have spurred much progress and debate in ecology during recent decades (Loreau *et al*. 2001; Hooper *et al*. 2012). These BEF studies provide the theoretical basis for the serious concern whether losses of biodiversity may diminish ecosystem functioning (e.g. productivity) and services (Cardinale *et al*. 2012). In terrestrial ecology a general consensus has been reached that, even after controlling for other confounding variables such as biomass, the effect of plant diversity, mostly represented by species richness (i.e. number of species), on primary productivity is generally positive, affirming the importance of protecting biodiversity (Cardinale *et al*. 2006, 2007; Tilman *et al*. 2014; Grace *et al*. 2016).

Positive relationships between diversity and productivity can arise from both ‘selection effects’ and ‘niche complementarity’ (Loreau & Hector 2001; Cardinale *et al*. 2006; Loreau 2010). Selection effects may enhance the productivity of more diverse communities when these have greater probability of including the most productive, i.e. best adapted, species that tend to dominate over time, compared to less diverse communities. The complementarity effect arises when niche differentiation confers complementary resource requirements to different species at different times or places, or when species coexistence is mutually beneficial via niche facilitation (e.g. symbiosis).

While the above insights have provided the theoretical basis for understanding the effects of diversity on productivity, the roles of environmental variability have not been thoroughly investigated. Theoretical studies have proposed that more diverse communities can be more productive in sufficiently variable environments, although the presence of unproductive species may reduce productivity under low variability (Norberg *et al*. 2001; Smith *et al*. 2016).

Here we investigate BEF relationship for marine phytoplankton, the most numerous photosynthetic organisms on Earth, which contribute nearly half of global primary production (Field *et al*. 1998). Pelagic ocean ecosystems differ fundamentally from their terrestrial counterparts. One obvious difference is that the dominant oceanic primary producers are phytoplankton, mostly smaller than 200 microns in diameter. BEF studies on marine microbial organisms that directly manipulate biodiversity are particularly scarce (Hooper *et al*. 2005; Krause *et al*. 2014). Some pioneering studies have used ocean numerical models to evaluate the effects of phytoplankton functional diversity on productivity (Goebel *et al*. 2014; Vallina *et al*. 2017). Their approach, similar to most BEF experiments, is to sample randomly subsets of different numbers of species from the total species pool and seed them into the same environment. The community is then allowed to self‐organise in the model ocean, and the results tend to confirm that species richness enhances productivity, as found in terrestrial BEF studies.

However, for microbial organisms it is widely accepted that, ‘Everything is everywhere, but the environment selects’ (Baas‐Becking 1934; Finlay 2002; Follows & Dutkiewicz 2011). That tenet suggests that for microbes, species richness can be practically infinite, which echoes with the familiar ‘Paradox of Plankton’ (Hutchinson 1961; Kashtan *et al*. 2014). Therefore, it is more appropriate to assume a continuous distribution for phytoplankton traits, here denoted by *l*. The community average growth rate can then be expressed in terms of the statistics of the trait distribution (Wirtz & Eckhardt 1996; Norberg *et al*. 2001; Merico *et al*. 2009):

(1a) $$\mu_{\mathrm{com}}={\left.\left(\mu +\frac{v}{2}\frac{d^{2}\mu }{dl^{2}}\right)\right|}_{l=\overset{¯}{l}}$$

(1b) $$\frac{d\overset{¯}{l}}{\mathrm{dt}}=v{\left.\frac{d\mu }{\mathrm{dl}}\right|}_{l=\overset{¯}{l}\;}$$

(1c) $$\frac{\mathrm{dv}}{\mathrm{dt}}=v^{2}{\left.\frac{d^{2}\mu }{dl^{2}}\right|}_{l=\overset{¯}{l}\;}$$

Here μ_com_ represents the per capita growth rate (d^−1^) of the total community, equivalent to $\frac{\mathrm{dP}}{\mathrm{Pdt}}$ where *P* is total community biomass of phytoplankton. Thus, μ_com_ can be an index for productivity, which is usually correlated with the formal definition of primary production (i.e. organic carbon produced per unit time) (Vallina *et al*. 2014b). In terrestrial studies, biomass yield is often used as a proxy for productivity. However, the fast turnover of oceanic phytoplankton may decouple their productivity from biomass.

$\overset{¯}{l}$ represents the mean of trait value *l*, which determines the per capita growth rate or fitness μ. *v* represents the variance of *l*, a proxy of diversity. The second derivative of μ ($\frac{d^{2}\mu }{dl^{2}}{|}_{l=\overset{¯}{l}}$), appearing in both eqn 1a and 1c can be understood as the effect of competition on both community productivity and diversity. Intense competition leads to narrow peaks of fitness around the optimal trait and reduces both productivity and diversity, known as ‘Competitive exclusion’. Eqn 1b states that the rate of change of mean trait $\overset{¯}{l}$ is proportional to trait diversity, analogous to Fisher's fundamental theorem of natural selection (Fisher 1930). Eqn 1b also captures the selection effect described above: greater diversity allows the community to retain more species that differ in some functionality, thereby enhancing community productivity under environmental fluctuation, known as the ‘insurance effect’ (Yachi & Loreau 1999). Therefore, eqn 1 provides an ideal theoretical framework for investigating BEF relationships for microbial organisms having nearly continuous trait distributions. Note that eqn 1 can be easily extended to two or more traits (Wirtz & Eckhardt 1996; Savage *et al*. 2007). For the sake of simplicity, we herein assume that size is the only master trait for phytoplankton, because many key traits that quantify aspects of phytoplankton physiology, such as nutrient uptake and photosynthesis, vary systematically with size (Litchman & Klausmeier 2008; Finkel *et al*. 2010; Edwards *et al*. 2012, 2015; Marañón 2015). Phytoplankton size structure is also an important determinant of community respiration (Del Giorgio & Williams 2005) and the efficiency of the biological pump, i.e. carbon export from the euphotic zone (Laws *et al*. 2000). We assume a trade‐off between maximal growth and adaptation to low resource (nutrient or light) availability. Small phytoplankton can be considered as ‘gleaners’ adapted to oligotrophic environments, while fast‐growing intermediate‐size ‘opportunists’ thrive in resource‐rich environments (Grover 1990; Barton *et al*. 2010; Smith *et al*. 2016; Vallina *et al*. 2017). Therefore, we expect this tradeoff to result in a complementarity effect in temporally and spatially variable environments, leading to an overall positive effect of size diversity on productivity (Loreau 2010). This community‐based approach is also computationally advantageous compared to resolving discretely a finite number of species. It therefore allows a wider range of set‐ups and numerical experiments compared to species‐based approaches.

Here we investigate the effects of phytoplankton size diversity on primary productivity in the North Pacific using the continuous trait‐based approach described above. We model the dynamics of total phytoplankton biomass, mean size (log cell volume) and size variance in a three‐dimensional ocean circulation model using realistic physical forcing for the North Pacific, covering from equatorial to subarctic regions. The North Pacific can be broadly classified into several major biogeographic provinces (Ducklow 2003; Moore *et al*. 2013). In the oligotrophic gyre, permanent stratification limits upward supply of nitrogen into the euphotic zone and therefore primary production remains low. In the subarctic North Pacific, characterised by low temperature and light, nitrate concentrations remain consistently high and primary production is potentially limited by iron availability. The equatorial Pacific is another high‐nitrogen‐low‐chlorophyll (HNLC) region where equatorial upwelling maintains high nitrate concentrations and production is limited by iron. Due to the growth limitations by either nitrogen, iron, or light, picophytoplankton (smaller than 3 μm in diameter) tend to dominate in the open ocean, while larger cells can become important in coastal waters (Odate 1996; Fujiki *et al*. 2014).

It needs to be emphasised that the complicated intertwining of diversity, productivity, and the environment in nature as well as in our model ocean poses a major challenge for BEF studies (Huston 1997; Grace *et al*. 2016). A significant bivariate correlation between productivity and diversity does not constitute unambiguous evidence for a positive or negative BEF relationship. To generate a diversity gradient independent of environmental effects, we employ two approaches to sustain different levels of diversity in the model. The first is to vary the ‘trait diffusion’ (TD) coefficient (*u*), which is the probability that the offspring of individuals from one trait class evolve into other trait classes via genetic mutation or trans‐generational plasticity (Merico *et al*. 2014). The second is to vary the zooplankton ‘kill‐the‐winner’ (KTW) grazing coefficient (*a*_*g*_), which describes how the zooplankton feeding preference changes with prey abundance (Vallina *et al*. 2014a,b, 2017; Wirtz 2014). By choosing different values of the TD and KTW parameters respectively, to generate diversity gradients, we can indirectly separate environmental effects on phytoplankton productivity from the effects of size diversity *per se* (Grace *et al*. 2016). By combining three‐dimensional (3D) ocean modelling and idealised simulation experiments, we test the following hypotheses:

1. Higher diversity (induced by mutation rates or density‐dependent feeding preferences) should lead to higher primary production in general.
2. The effects of size diversity on productivity should depend on which environmental factor (nitrogen, iron or light) is most limiting for phytoplankton growth.
3. The two approaches that resolve discrete species vs. moments of the continuous distribution should generate consistent patterns.

## Materials and Methods

### North Pacific model

We constructed a plankton ecosystem model within which phytoplankton size follows a continuous distribution and then coupled the ecosystem model with a three‐dimensional hydrodynamic model of the North Pacific (Shchepetkin & McWilliams 2005). The ecosystem model was built on a typical nitrogen‐based, Nutrient‐Phytoplankton‐Zooplankton‐Detritus (NPZD) plankton model with the addition of an iron cycle and a lognormal distribution for phytoplankton size (cell volume) (see Fig. S1 in Supporting Information). We quantify size diversity in terms of the variance of log‐transformed cell volume, following previous studies (Wirtz 2014; Acevedo‐Trejos *et al*. 2015; Smith *et al*. 2016). The model details have been reported in Chen & Smith (2018) with the only difference that only one zooplankton compartment is included in the present study. Here we briefly describe the main features that are most relevant for the present study. The ecosystem model has eight tracers (dissolved inorganic nitrogen (*N*, mmol N m^−3^), phytoplankton (*P*, mmol N m^−3^), zooplankton (*Z*, mmol N m^−3^), detrital nitrogen (*D*, mmol N m^−3^), dissolved iron (*fer,* μmol m^−3^), detrital iron (*DETFe*, μmol m^−3^) and two raw moments of phytoplankton biomass distribution ($P\overset{¯}{l}$ and $P({\overset{¯}{l}}^{2}+\nu )$ where $\overset{¯}{l}$ is mean log volume (μm^3^) and ***v*** is the variance ((log μm^3^)^2^) of log volume)). Bruggeman (2009) has shown that the raw moments of the phytoplankton biomass distribution can be treated as typical tracers subject to advection and diffusion.

For a given size class, phytoplankton per capita growth rate (μ, d^−1^) depends on temperature, light (*I*, W m^−2^), *N* and *fer*, following the Liebig Monod‐type function:

(2) $$\mu \left(l,N,fer,I\right)=\mu_{m}\min\left(\frac{N}{N+K_{N}},\frac{\mathrm{fer}}{fer+K_{\mathrm{fer}}}\right)\left(1-e^{-\frac{\alpha_{c}I}{\mu_{m}}}\right)$$

with all the parameters including maximal growth rate (μ_*m*_), half‐saturation constant for nitrogen (*K*_*N*_), initial slope for the photosynthesis‐irradiance curve (*a*_*c*_) and half‐saturation constant for iron (*K*_*fer*_) depending on cell size:

(3a) $$\mu_{m}=\mu_{0}e^{\alpha_{\mu }l+\beta_{\mu }l^{2}}$$

(3b) $$K_{N}=K_{0,N}e^{\alpha_{K}l}$$

(3c) $$K_{\mathrm{fer}}=K_{0,fer}e^{\alpha_{\mathrm{fer}}l}$$

(3d) $$\alpha_{c}=\alpha_{0,c}e^{\alpha_{I}l}$$

where μ_0_, *K*_*0*,*N*_, *K*_*0*,*fer*_, *a*_*0*,*c*_, *a*_μ_, β_μ_, *a*_*K*_, *a*_*fer*_ and *a*_*I*_ are parameters independent of size (Table S1). Here μ_*m*_ is assumed as a unimodal function of *l*, reflecting higher respiratory costs in picoplankton, which gives nanoplankton an advantage under nutrient‐replete conditions (Chen & Liu 2010; Wirtz 2011; Marañón *et al*. 2013). The half‐saturation constants increase with size, which favours small sizes in oligotrophic environments. Hence, this parameterisation constitutes a trade‐off between maximal growth and adaptation to low nutrient availability for marine pico‐ and nano‐phytoplankton that are the dominant primary producers in the open ocean. Compared to nutrients, the effect of light on size is weaker (i.e. *a*_*I*_ < *a*_*K*_), which still confers some advantage to small phytoplankton under light limitation (Edwards *et al*. 2015).

Carbon based net primary production (NPP, mg C m^−3^ d^−1^) on the community level is calculated as:

(4) $$NPP={\left.P\left(\frac{\mu }{Q_{N}}+\frac{v}{2}\frac{\partial^{2}\left(\frac{\mu }{Q_{N}}\right)}{dl^{2}}\right)\right|}_{l=\overset{¯}{l}\;}$$

in which *Q*_*N*_ is the nitrogen‐to‐carbon ratio (mol N (mol C)^−1^) that increases with ambient *N* or *fer* (assuming iron availability limits nitrogen uptake under iron limitation; Morel 1987):

(5) $$Q_{N}=\frac{Q_{\min}}{1-\left(1-\frac{Q_{\min}}{Q_{\max}}\right)\min\left(\frac{N}{N+K_{N}},\frac{\mathrm{fer}}{fer+K_{\mathrm{fer}}}\right)}$$

where *Q*_min_ and *Q*_max_ are the minimal and maximal nitrogen‐to‐carbon ratios respectively.

Further model details relevant to size diversity, the TD coefficient *u* and the KTW coefficient *a*_*g*_, are given in the Supporting Online Materials and Chen & Smith (2018). To analyse the effect of the diversity enhancing coefficients on total NPP, we also decomposed the differences of integrated NPP among model runs using the chain rule:

(6) $$\begin{array}{cc} \frac{\Delta \int NPPdVdt}{\Delta u} & =\int \left\{\frac{\mu }{Q_{N}}\frac{\Delta P}{\Delta u}+\frac{P}{Q_{N}}\frac{\Delta \mu }{\Delta u}-\frac{P\mu }{{Q_{N}}^{2}}\frac{\Delta Q_{N}}{\Delta u}\right.\\ & \left.\;+\frac{1}{2}\left[vP\frac{\Delta \left(\frac{\partial^{2}\left(\frac{\mu }{Q_{N}}\right)}{\partial l^{2}}\right)}{\Delta u}+v\frac{\partial^{2}\left(\frac{\mu }{Q_{N}}\right)}{\partial l^{2}}\frac{\Delta P}{\Delta u}+P\frac{\partial^{2}\left(\frac{\mu }{Q_{N}}\right)}{\partial l^{2}}\frac{\Delta v}{\Delta u}\right]\right\}dVdt \end{array}$$

where ∫NPPdVdt is the NPP integrated over all model grids (*dV* stands for the volume of each grid) from 0 to 260 m over an annual cycle. $\frac{\Delta P}{\Delta u}$ means the differences of the quantity *P* normalized by the differences in the TD or KTW coefficient *u*.

To further understand the contributions of various factors affecting the long‐term average (i.e. expectation) of phytoplankton growth rate at the mean size (eqn (2)), which is the major quantity affecting NPP (see Results), in a seasonally variable environment, we applied the technique of Taylor expansion around the seasonal mean to the second‐order (Wirtz 2000; Mandal *et al*. 2014):

(7) $$\begin{array}{cc} \bar{\mu \left(\overset{¯}{l},N,I\right)} & =\mu \left({\overset{¯}{l}\;}_{0},N_{0},I_{0}\right)+\frac{1}{2}\left[{\left.\frac{\partial^{2}\mu }{\partial {\overset{¯}{l}\;}^{2}}\right|}_{\overset{¯}{l}\;={\overset{¯}{l}\;}_{0}}\sigma_{\overset{¯}{l}\;}^{2}\right.\\ & \;\left.+{\left.\frac{\partial^{2}\mu }{\partial N^{2}}\right|}_{N=N_{0}}\sigma_{N}^{2}+{\left.\frac{\partial^{2}\mu }{\partial I^{2}}\right|}_{I=I_{0}}\sigma_{I}^{2}\right.\\ & \;\left.+2{\left.\frac{\partial^{2}\mu }{\partial \overset{¯}{l}\;\partial N}\right|}_{\overset{¯}{l}\;={\overset{¯}{l}\;}_{0,}N=N_{0}}\sigma_{\overset{¯}{l}\;N}+2{\left.\frac{\partial^{2}\mu }{\partial \overset{¯}{l}\;\partial I}\right|}_{\overset{¯}{l}\;={\overset{¯}{l}\;}_{0,}I=I_{0}}\sigma_{\overset{¯}{l}\;I}\right.\\ & \;\left.+2{\left.\frac{\partial^{2}\mu }{\partial I\partial N}\right|}_{I=I_{0,}N=N_{0}}\sigma_{\mathrm{IN}}\right] \end{array}$$

in which ${\overset{¯}{l}}_{0}$, *N*_0_ and *I*_0_ represents the annual mean phytoplankton mean size ($\overset{¯}{l}$), limiting nutrient *N* (nitrogen or iron), and light level (*I*) respectively, in each model grid. $\sigma_{x}^{2}$ and $\sigma_{\mathrm{xy}}$ represent the temporal variance of *x* and covariance between *x* and *y* (*x* or *y* represents $\overset{¯}{l}$, *N* or *I*) respectively. The first term on the right side can be treated as a constant. Because our model results show that the seasonal variations of light, nitrate and iron are relatively insensitive to the coefficients (*u*) of TD or KTW (data not shown), the effect of *u* on the long‐term expectation of phytoplankton growth rate can be simplified to:

(8) $$\frac{\Delta \overset{¯}{\mu }\;}{\Delta u}=\frac{1}{2}{\left.\frac{\partial^{2}\mu }{\partial {\overset{¯}{l}\;}^{2}}\right|}_{\overset{¯}{l}\;={\overset{¯}{l}\;}_{0}}\frac{\Delta \sigma_{\overset{¯}{l}\;}^{2}}{\Delta u}+{\left.\frac{\partial^{2}\mu }{\partial \overset{¯}{l}\;\partial N}\right|}_{\overset{¯}{l}\;={\overset{¯}{l}\;}_{0,}N=N_{0}}\frac{{\Delta \sigma }_{\overset{¯}{l}\;N}}{\Delta u}+{\left.\frac{\partial^{2}\mu }{\partial \overset{¯}{l}\;\partial I}\right|}_{\overset{¯}{l}\;={\overset{¯}{l}\;}_{0,}I=I_{0}}\frac{{\Delta \sigma }_{\overset{¯}{l}\;I}}{\Delta u}$$

We varied the values of TD or KTW coefficients from 0 to 0.1 to generate six diversity gradients (*u* or *a*_*g*_ = 0, 0.01, 0.03, 0.05, 0.07, 0.1) so that a total of 11 simulations were run in three‐dimension. After each simulation was run for 10 years, the total annual NPP integrated throughout the euphotic zone (from surface to 260 m) over the entire model domain was calculated for the final year.

### Idealised simulation experiments

We conducted idealised simulation experiments to compare our continuous size‐based approach with the conventional approach of controlling the level of species richness, based on a simple phytoplankton‐nutrient model that has often been applied to investigate the effects of environmental variability on species coexistence (Supporting Online Material; Huisman 2010; Loreau 2010). For both models, the external nutrient supply varied as a seasonal sinusoidal function. Two amplitudes of the sinusoidal function were set up to simulate different levels of environmental variability. For the continuous model, we fixed the size diversity at different levels and, for each diversity level, took a random value between −2.73 log μm^3^ and 15.2 log μm^3^ (equivalent to 0.5 and 200 μm in diameter) as the initial mean size (log cell volume) of the community, running a total of 50 replicate simulations at each diversity level. In the case of controlling species richness (*n*, 1 ≤ *n* ≤ 10) with the discrete model, at each richness level, we sampled *n* species with sizes randomly distributed between −2.73 log μm^3^ and 15.2 log μm^3^ and ran 50 replicates. Each model configuration was run for 10 years and the annual NPP was calculated for the final year.

## Results

**Figure 1:**
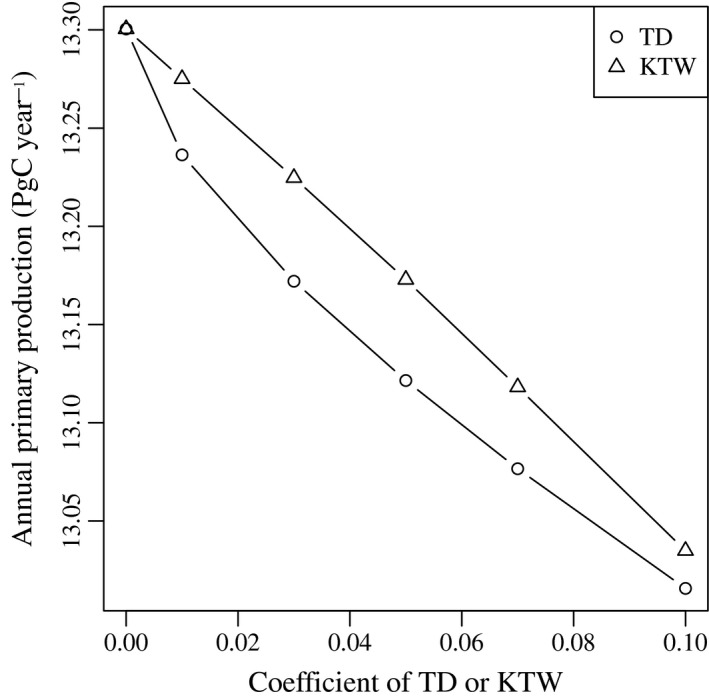
Modelled annual primary production integrated for the euphotic zone (0–260 m) summed over the whole model domain against the coefficients of ‘trait diffusion’ or ‘kill‐the‐winner’.

The model was able to reproduce the large‐scale patterns of nitrate, chlorophyll *a* and NPP (Fig. S2). Total NPP over our model domain was estimated between 13.0 and 13.3 Pg year^−1^ (Fig. 1), roughly one‐fourth of annual NPP for the global ocean (Field *et al*. 1998). Total NPP was inversely related to *u* and *a*_*g*_, although the variations were negligible. Total NPP was 2% higher in the lowest diversity treatment (*u* = 0 and *a*_*g*_ = 0) compared with the highest diversity treatments (*u* = 0.1 or *a*_*g*_ = 0.1).

**Figure 2:**
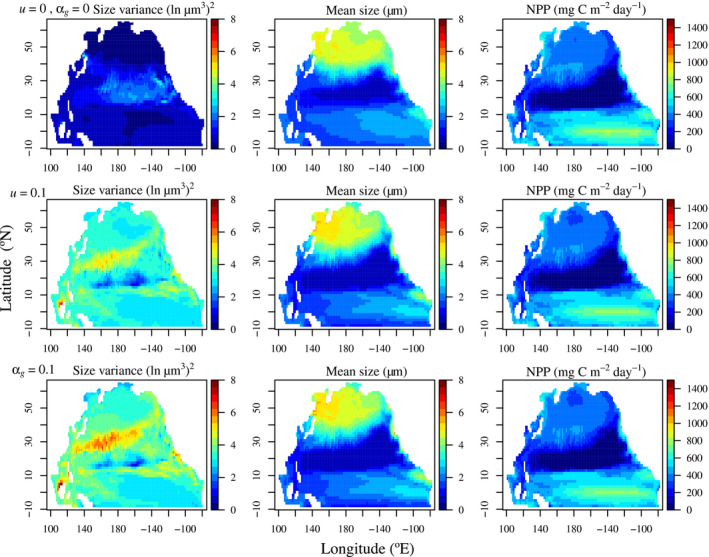
Modelled annual mean patterns of size diversity, mean size and primary production (NPP) for three diversity treatments. The first row is the lowest diversity treatment without any TD or KTW. The second and third rows are the treatments with the largest TD and KTW coefficients.

The spatial patterns of annual mean NPP confirmed the negligible differences between the highest (*u* = 0.1 or *a*_*g*_ = 0.1) and the lowest (*u* = *a*_*g*_ = 0) diversity treatments, although, as expected, size diversity was substantially lower in the lowest diversity treatment than in the highest diversity treatment (Fig. 2). The mean phytoplankton sizes and nitrogen biomass were similar among all treatments.

**Figure 3:**
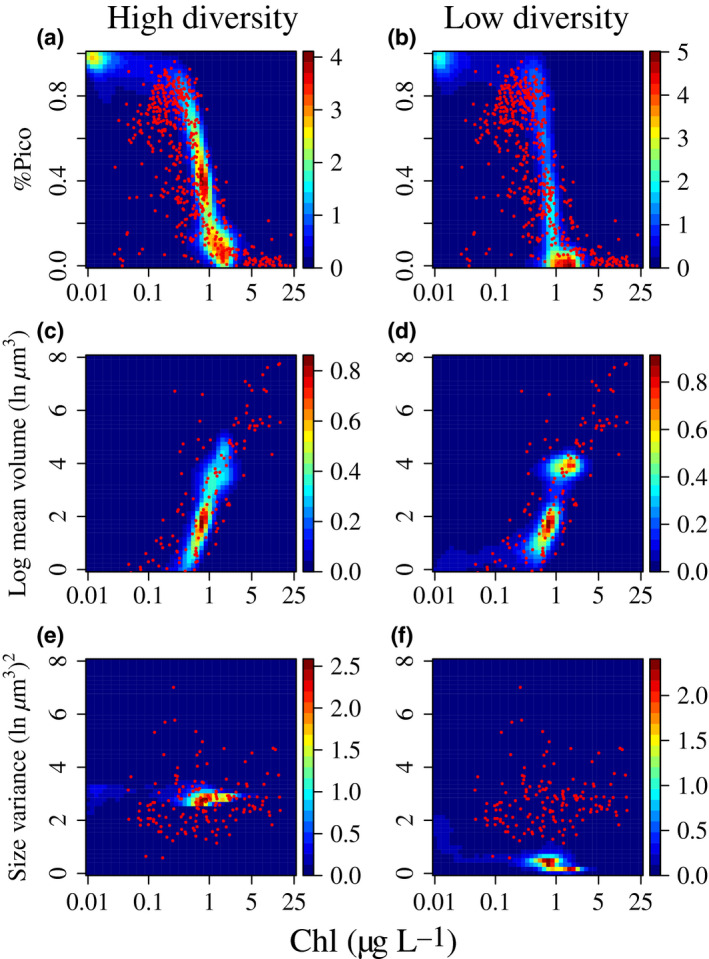
Simulated patterns of (a,b) fractions of picophytoplankton (< 2 μm), (c,d) mean size and (e,f) size variance of high (*u* = 0.1) and low diversity treatments. Colour contours indicate model data density. Red dots indicate observational data from Marañón *et al*. (2012).

Compared to the lowest diversity treatment, the highest diversity treatment agreed better with observed size‐fractionated chlorophyll patterns in terms of picophytoplankton (< 2 μm) and size variance, while both treatments similarly matched mean size data (Fig. 3). With no TD or KTW to sustain diversity, the model severely underestimated size variance (Fig. 3c).

To understand why enhancing size diversity by TD or KTW has negligible effects on NPP, we calculated and integrated the components in eqn (6) for the lowest and highest diversity treatments respectively (Table 1). The three most important quantities were the differences of growth rate $\mu (\overset{¯}{l})$, size diversity and nitrogen‐to‐carbon ratios (*Q*_N_) at mean (i.e., dominant) size respectively. However, the positive effect of size diversity on growth $\mu (\overset{¯}{l})$ induced by high mutation rate (*u*) was overall counterbalanced by the negative effect of including more unproductive species (i.e. higher *v*), because $\frac{\partial^{2}\left(\frac{\mu }{Q_{N}}\right)}{\partial l^{2}}$ was usually negative at mean size. The third factor, *Q*_N_, eventually determined the negative, albeit small, effect of size diversity on NPP. This is because high *Q*_N_ was usually associated with high growth rate $\mu (\overset{¯}{l})$, reducing organic carbon production per unit nitrogen biomass. That is, although higher diversity tended to enhance $\mu (\overset{¯}{l})$, it also enhanced *Q*_*N*_, which counteracted the effect of diversity on NPP.

**Table 1:** Decompositions of contributions of different components (unit: Pmol C year^−1^ d (ln μm^3^)^2^) in eqn (6)

$\int \frac{P}{Q_{N}}\frac{\Delta \mu }{\Delta u}dVdt$	4.6
$-\int \frac{P\mu }{{Q_{N}}^{2}}\frac{\Delta Q_{N}}{\Delta u}dVdt$	−2.8
$\int \frac{\mu }{Q_{N}}\frac{\Delta P}{\Delta u}dVdt$	−0.12
$\frac{\int vP\frac{\Delta \left(\frac{\partial^{2}\left(\frac{\mu }{Q_{N}}\right)}{\partial l^{2}}\right)}{\Delta u}dVdt}{2}$	−0.07
$\frac{\int v\frac{\partial^{2}\left(\frac{\mu }{Q_{N}}\right)}{\partial l^{2}}\frac{\Delta P}{\Delta u}dVdt}{2}$	−0.03
$\frac{\int P\frac{\partial^{2}\left(\frac{\mu }{Q_{N}}\right)}{\partial l^{2}}\frac{\Delta v}{\Delta u}dVdt}{2}$	−4.6

**Figure 4:**
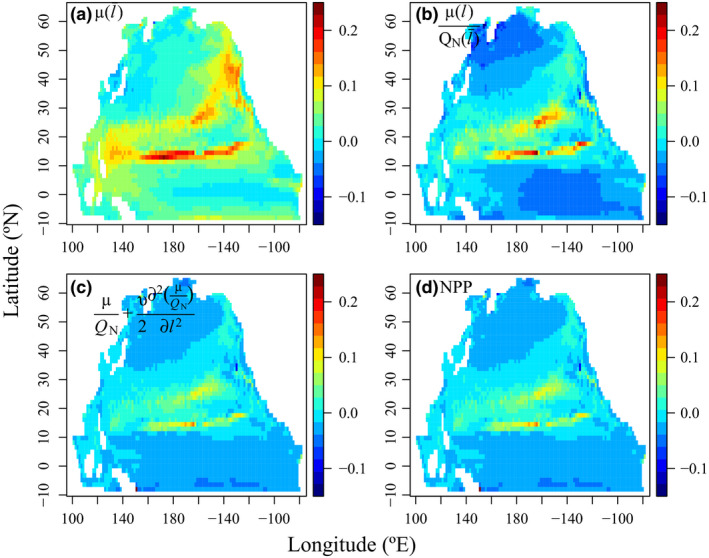
Spatial patterns of log ratios of annual mean (a) phytoplankton growth rate at mean size, (b) carbon‐based growth rate at mean size, (c) carbon‐based phytoplankton growth rate of the whole community and (d) carbon‐based primary production (NPP) integrated from surface to 260 m in the diversity treatments with the highest vs. the lowest coefficients of trait diffusion.

We examined the spatial patterns of the effects of TD coefficient *u* on phytoplankton growth rates and NPP by calculating the logarithmic ratios of the annual $\mu (\overset{¯}{l})$, carbon‐based growth rate $\frac{\mu }{Q_{N}}{|}_{l=\overset{¯}{l}}$, community average carbon‐based growth rate $\frac{\mu }{Q_{N}}+\frac{v}{2}\frac{\partial^{2}\left(\frac{\mu }{Q_{N}}\right)}{\partial l^{2}}{|}_{l=\overset{¯}{l}}$ and NPP, each from the highest (*u* = 0.1) to the lowest diversity treatment (*u* = 0) and integrated throughout the euphotic zone (Fig. 4). The positive effect of size diversity on $\mu (\overset{¯}{l})$, which prevails over most regions, is strongest along the fronts between the central oligotrophic regions and the two adjoining subarctic and equatorial regions (Fig. 4a). In regions where light and iron limitations are more important (Fig. S4), size diversity only weakly affects $\mu (\overset{¯}{l})$. The effect of *Q*_N_ weakens the net effect of size diversity on carbon‐based growth rate (Fig. 4b). Further considering the effect of unproductive species within the community (i.e. the effect of *v*), we found that the positive effect of size diversity on community‐integrated carbon‐based growth rate was reduced more. However, in the subarctic and equatorial Pacific, where light limitation was strongest (Fig. S4), this decrease was absent or even reversed (Fig. 4c). Because the summed negative effects overweighed the summed positive effects, the net effect of size diversity on NPP was slightly negative for the whole North Pacific (Fig. 4d).

**Figure 5:**
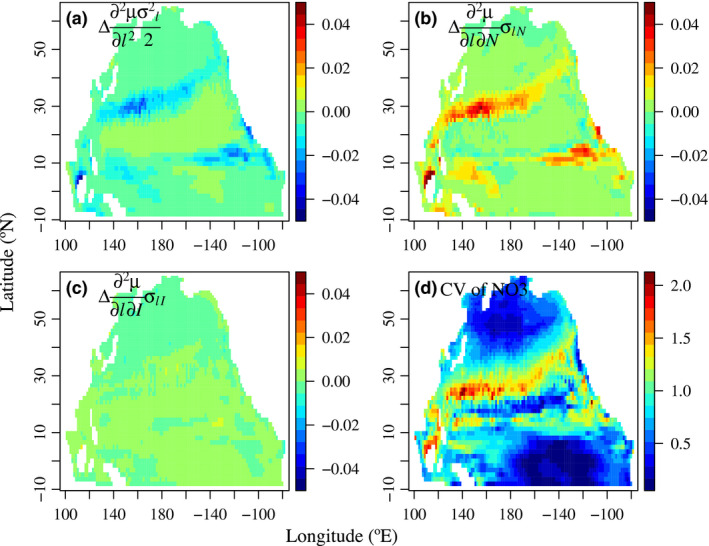
Spatial patterns of (a,b,c) differences of different components of R.H.S. of eqn (8) including seasonal variances of mean size and covariances between mean size and light/nutrient in surface waters between the high‐ and low‐diversity treatments. (d) Coefficients of variation (= SD/Mean) of surface nitrate concentrations.

To better understand the mechanisms of diversity effects on phytoplankton growth rate $\mu (\overset{¯}{l})$, we plotted the spatial patterns of the three components of the right side of eqn (8) within the surface mixed layer, as their difference between the high and low diversity treatments (Fig. 5). The contributions of $\sigma_{\overset{¯}{l}}^{2}$ to $\mu (\overset{¯}{l})$ differences were negative along the fronts separating the central gyre and the adjoining north and south areas, while the contributions of $\sigma_{\overset{¯}{L}N}$ (covariance between mean size and nutrient) were positive, corresponding to the coefficients of variations of surface nitrate. The effects of $\sigma_{\overset{¯}{L}I}$ (covariance between mean size and light) were relatively minor.

**Figure 6:**
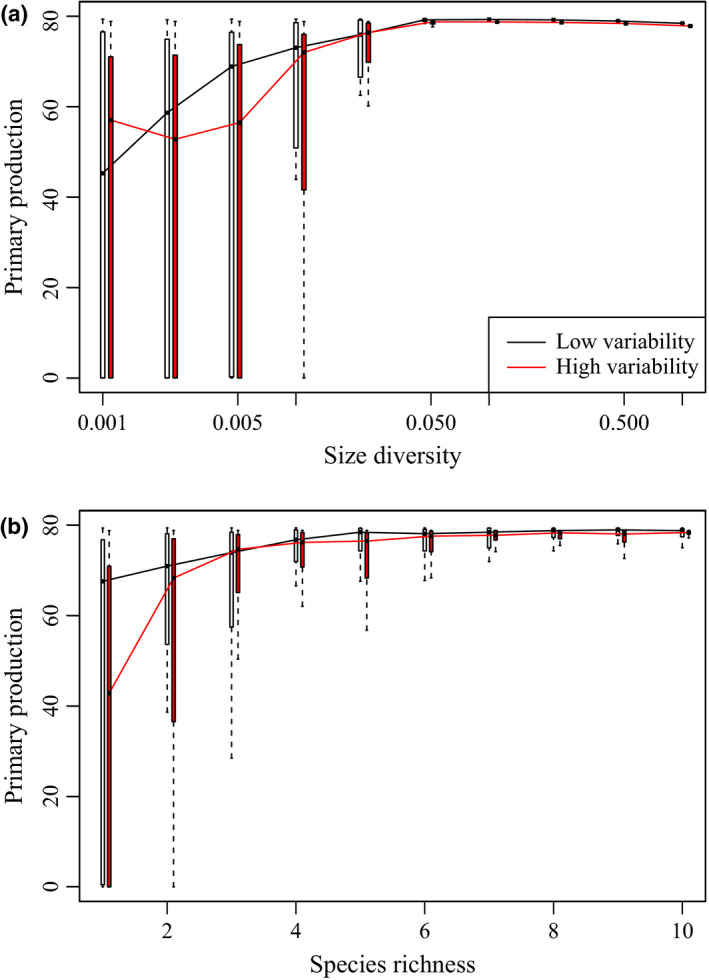
Simulated relationships between primary production and diversity using (a) continuous trait‐based approach that assumes a lognormal distribution for phytoplankton size and (b) discrete species approach. Imposed box‐and‐whisker plots show the median (thick lines), 25th and 75th percentiles (box edges), data ranges and outliers (open circles).

We ran a series of idealised simulation experiments to better understand the differences between our continuous trait‐based approach, in which size diversity is the key diversity metric and the more typical approach that takes species richness as the diversity metric. For both levels of environmental variability, we found that the median NPP increased from the lowest to an intermediate level of size diversity (0.05 (log μm^3^)^2^) and then slightly decreased with increasing size diversity afterwards (Fig. 6a). When we used the more typical approach of randomly sampling a given number of species, we obtained the familiar increasing trend of NPP with species richness, for which NPP reached a plateau beyond the richness of 4 (Fig. 6b; Goebel *et al*. 2014; Vallina *et al*. 2017). For both models, at low diversity levels, NPP values are higher under low environmental variability.

## Discussion

Our result that increasing size diversity has negligible or even negative effect on NPP across the whole North Pacific seems to contradict many previous reports of positive effects of species richness on ecosystem productivity (Cardinale *et al*. 2006, 2007; Tilman *et al*. 2014), including studies using ocean circulation models (Goebel *et al*. 2014; Vallina *et al*. 2017). This is somehow unexpected given the already well described theoretical mechanisms including both ‘selection effects’ and ‘niche complementarity’ and experimental evidences for the positive effect of diversity on productivity (Hooper *et al*. 2005; Cardinale *et al*. 2012; Tilman *et al*. 2014). However, as argued in Hillebrand & Matthiessen (2009), the understanding of diversity effects on productivity would be superficial if a causal linkage between individual functional traits and ecosystem functioning is not adequately set up. We propose that eqns (6), (7), (8) can be used as a quantitative framework to understand diversity effects on productivity in variable environments and then the specifics conditions for negligible or negative diversity effects on productivity to occur can be inferred.

### Covariance between trait and environment

Equation 1a reveals that the positive effect of diversity on productivity at the community level must counteract the generally negative effect of diversity on the growth rate at the mean (best‐adapted) trait, because the second derivative of growth rate $\left(\frac{\partial^{2}\mu }{\partial {\overset{¯}{l}}^{2}}{|}_{\overset{¯}{l}={\overset{¯}{l}}_{0}}\right)$ should be negative under most conditions. In the absence of positive species interactions or niche differentiation under static conditions as in our case, diversity can enhance productivity exclusively in a variable environment. More specifically, based on eqn (8), only the covariance between the trait and the environment can contribute to positive effects of diversity on productivity. The positive effect of size diversity on productivity under environmental variability involves a selection effect. Compared to a low diversity community, in a high diversity community the dominant trait class tracks the environment faster, i.e. approaches closer to the optimal trait (Fig. S6). Favourable environments (high nutrient or light) select fast growing opportunists of large size (within the phytoplankton size spectrum relevant for the North Pacific), while unfavourable environments select small gleaners that are relatively insensitive to resource shortage. This temporal or spatial niche partitioning may also be viewed as a result of spatiotemporal complementarity increasing overall production, analogous to the ‘storage effect’ induced by the covariance between the environment and competition, which helps to maintain diversity in variable environments (Chesson 2000).

### Importance of trait characteristics

Equation (8) makes clear that different functional growth dependencies, on both traits and environmental conditions, can have different effects on BEF relationships. For example, light can affect both phytoplankton size and productivity. The covariance between light and mean size ($\sigma_{\overset{¯}{l}I}$) turns out to be less important than $\sigma_{\overset{¯}{l}N}$ because the combined growth dependency $\frac{\partial^{2}\mu }{\partial \overset{¯}{l}\partial I}{|}_{\overset{¯}{l}={\overset{¯}{l}}_{0},I=I_{0}}$ is small. Therefore, despite sometimes large differences of $\sigma_{\overset{¯}{l}I}$ between different diversity treatments, the overall light effect on productivity is relatively small compared to the nutrient effects (Figs S7 and S8). This is because most production takes place within the surface mixed layer with relatively high light but low nutrient availability. Furthermore, the optimal size class at low nutrient levels is similar to that under light limitation, so that moderate changes in light levels hardly affect the size distribution.

Loreau (2010) emphasised that the asynchronous responses of species in variable environments are the key to temporal complementarity, which generates a positive diversity effect on productivity. By contrast, synchronised responses can be understood as niche overlapping. This implies that niche differentiation can promote diversity maintenance and positive diversity effects on productivity, particularly under environmental variability. Vallina *et al*. (2017) pointed out that the complementarity effect may be negligible for niches with open‐ended forms such as nutrient uptake; i.e. nutrient uptake is not inhibited at high concentrations. This is because even in the absence of the most productive species, a less efficient species can still occupy its niche (utilize the nutrients) at a rate only slightly slower. In the present study, both nutrient uptake and light acquisition are open‐ended functions with positive first derivatives and negative second derivatives. Species responses to both nutrient or light are more synchronised at high resource levels, typical of light, than at low resource levels, typical of nutrient, making nutrient more important than light for determining diversity‐productivity relationships. Much stronger complementarity effects are expected for closed‐ended niches (e.g. unimodal functions) such as optimal temperature or light, which impose greater disadvantages on sub‐optimal species (Vallina *et al*. 2017). That is, on a continuous trait space, fitness decreases more steeply away from the optimal trait for a closed‐ended niche than for an open‐ended one (fig. 2 in Vallina *et al*. 2017). Hence for a closed‐ended niche in a dynamic environment, greater trait variance/diversity *v* is more likely to enhance fitness (up to a point), by increasing the speed at which the mean trait (eqn 1b) tracks its optimal value, resulting in stronger covariance between trait and environment.

The master trait cell size mostly correlates with nutrient uptake strategies (Litchman *et al*. 2007; Edwards *et al*. 2012; Marañón *et al*. 2013). Thus, similar to nutrient uptake traits, the lack of a strong complementarity effect of size diversity on productivity is therefore not surprising. For future studies it would certainly be worthwhile to include more traits, such as optimal growth temperature and light that are not strongly dependent on size, particularly in areas with substantial seasonal or spatial fluctuations of temperature or light (Goebel *et al*. 2014; Vallina *et al*. 2017; Bestion *et al*. 2018).

### Environmental variability and negative effect of trait diversity on productivity

Since early BEF studies, it has already been realised that at high diversity levels, increasing diversity can reduce productivity particularly when the environment is not sufficiently dynamic, which can lead to a unimodal relationship between productivity and diversity (Hector *et al*. 1999; Norberg *et al*. 2001; Hillebrand & Matthiessen 2009; Tilman *et al*. 2014; Smith *et al*. 2016). As such, it is not surprising to find negative effects of size diversity on community productivity, which usually occur in areas with negligible $\sigma_{\overset{¯}{l}N}$ and surface nitrate varies little (Figs 4 and 5). Conversely, in dynamic environments, high diversity is needed to achieve selection effects sufficiently strong to generate positive covariance between the mean size (trait) and nitrate (environment) and thereby to enhance productivity (Figs 5 and 6).

### Saturating diversity levels for productivity

Schwartz *et al*. (2000) pointed out that productivity may saturate at quite low diversity levels, less than half of total species richness. Our idealised simulation experiments showed that the positive effect of size diversity on productivity is evident only at very low levels of size diversity (< 0.05 (ln μm^3^)^2^), above which this effect becomes negligible (Fig. 5). This effect is also evident in the discrete case, in which productivity saturates around richness of 4. The low level of size diversity that saturates productivity also relates to the trait characteristics (e.g. the open‐ended niche form) discussed above.

In the 3D model ocean, even in the unrealistic cases without TD or KTW to sustain diversity, advection and diffusion still maintain levels of size diversity greater than 0.05 (ln μm^3^)^2^ in many areas (Figs 2 and 3). Vertically, deepening mixed layers during fall can entrain communities living at depth where nutrients are plentiful but light is limiting, into surface waters where nutrient can be limiting (Chen & Smith 2018). Horizontally, high diversity can also emerge along ocean fronts where mixing is active (Barton *et al*. 2010). Thus, three‐dimensional water mixing can be an important mechanism to sustain diversity and consequently relatively high productivity. However, some additional mechanism such as TD or KTW is required to reproduce the observed levels of phytoplankton size diversity based on size‐fractionated chlorophyll measurements (Fig. 3). Therefore, we expect that within realistic ranges of TD or KTW parameters, the weak negative effect of size diversity on productivity should be robust.

### Stoichiometry effects

Another important factor contributing to the reduction of integrated carbon‐based NPP with increasing diversity is the variable nitrogen‐to‐carbon ratio of phytoplankton, which offsets the enhancement of growth rate at high diversity (Table 1). This underlines the importance of a clear definition of *productivity* (per capita growth rate) as distinct from absolute rates of *production*. Our results are consistent with the view that the emergent pattern of phytoplankton stoichiometry can result from diversity and, vice versa, stoichiometry can also affect competition and diversity patterns among phytoplankton (Bonachela *et al*. 2015). Classical competition theory has rarely accounted for flexible stoichiometry. Future studies are needed to extend the size‐based approach to multiple traits including flexible stoichiometry. This will be complicated, because minimal and possibly also maximal nutrient ratios correlate with size, while the actual C : N : P : Si : Fe : Chl ratios mostly reflect the environmental history of cells. Furthermore, diversity needs to be incorporated into a coherent and robust modelling framework that can be practically useful for assessing how global change and anthropogenic activities affect biodiversity and ecosystem functioning in the ocean.

### Concluding remarks

While our first assessment reveals a negligible effect of phytoplankton size diversity on productivity, the mechanisms underpinning BEF relationships should be common to all ecosystems. Particularly, our analysis suggests the importance of interactions between environmental variability and trait distributions. The *environment* can encompass several dimensions, such as light, temperature and different nutrients in the case of autotrophs and food concentrations and qualities in the case of heterotrophs. The effective *trait* space is therefore multi‐dimensional, with each dimension relating to one or more environmental axes (Savage *et al*. 2007). The details of each function relating traits and the environment may have far‐reaching implications for the intricate interactions among the environment, biodiversity and ecosystem functioning. Thus, it is expected that the overall diversity effect on productivity depends on the spatial scale considered, with higher probability of positive effect of diversity on productivity to be observed in environmentally more dynamic regions such as ocean fronts. While our exercise provides an initial step, better mathematical and modelling tools are needed to disentangle such complexity. On the experimental side, marine experiments similar to the terrestrial Cedar Creek and BIODEPTH experiments that examined the effects of diversity *per se* on productivity (Tilman *et al*. 1997; Hector *et al*. 1999) are also much needed to validate the results of theory and numerical experiments.

## Authorship

BC and SLS designed the study. BC wrote the model codes, ran the simulations, and wrote the first draft of the manuscript. All authors discussed the results and contributed to revisions.

## Conflict of Interest

The authors declare that they have no conflict of interest.

## Data Availability

The model and observational data are archived in https://github.com/BingzhangChen/ROMS-NPZDcont.git (https://doi.org/10.5281/zenodo.1410803).

## References

[ele13167-bib-0001] Acevedo‐Trejos, E. , Brandt, G. , Bruggeman, J. & Merico, A. (2015). Mechanisms shaping size structure and functional diversity of phytoplankton communities in the ocean. Sci. Rep., 5, 8918 10.1038/srep08918. 25747280 PMC5390085

[ele13167-bib-0002] Baas‐Becking, L.G.M. (1934). Geobiologie of inleiding tot de milieukunde. van Stockum and Zoon, The Hague.

[ele13167-bib-0003] Barton, A.D. , Dutkiewicz, S. , Flierl, G. , Bragg, J. & Follows, M.J. (2010). Patterns of diversity in marine phytoplankton. Science, 327, 1509–1511. 20185684 10.1126/science.1184961

[ele13167-bib-0004] Bestion, E. , García‐Carreras, B. , Schaum, C.E. , Pawar, S. & Yvon‐Durocher, G. (2018). Metabolic traits predict the effects of warming on phytoplankton competition. Ecol. Lett., 21, 655–664. 29575658 10.1111/ele.12932 PMC6849607

[ele13167-bib-0005] Bonachela, J.A. , Klausmeier, C.A. , Edwards, K.F. , Litchman, E. & Levin, S.A. (2015). The role of phytoplankton diversity in the emergent oceanic stoichiometry. J. Plankton Res., 38, 1021–1035.

[ele13167-bib-0006] Bruggeman, J. (2009). Succession in plankton communities: a trait‐based perspective. Ph.D. thesis, 160 pp.

[ele13167-bib-0007] Cardinale, B.J. , Srivastava, D.S. , Duffy, J.E. , Wright, J.P. , Downing, A.L. , Sankaran, M. *et al* (2006). Effects of biodiversity on the functioning of trophic groups and ecosystems. Nature, 443, 989–992. 17066035 10.1038/nature05202

[ele13167-bib-0008] Cardinale, B.J. , Wright, J.P. , Cadotte, M.W. , Carroll, I.T. , Hector, A. , Srivastava, D.S. *et al* (2007). Impacts of plant diversity on biomass production increase through time because of species complementarity. Proc. Natl Acad. Sci., 104, 18123–18128. 17991772 10.1073/pnas.0709069104 PMC2084307

[ele13167-bib-0009] Cardinale, B.J. , Duffy, J.E. , Gonzalez, A. , Hooper, D.U. , Perrings, C. , Venail, P. *et al* (2012). Biodiversity loss and its impact on humanity. Nature, 486, 59–67. 22678280 10.1038/nature11148

[ele13167-bib-0010] Chen, B. & Liu, H. (2010). Relationships between phytoplankton growth and cell size in surface oceans: Interactive effects of temperature, nutrients, and grazing. Limnol. Oceanogr., 55, 965–972.

[ele13167-bib-0011] Chen, B. & Smith, S.L. (2018). CITRATE 1.0: Phytoplankton continuous trait‐distribution model with one‐dimensional physical transport applied to the North Pacific. Geophys. Model Dev., 11, 467–495.

[ele13167-bib-0012] Chesson, P. (2000). Mechanisms of maintenance of species diversity. Ann. Rev. Ecol. Syst., 31, 343–366.

[ele13167-bib-0013] Del Giorgio P.A. & Williams P.J.L.B. (eds.) (2005). Respiration in Aquatic Ecosystems. Oxford University Press, New York.

[ele13167-bib-0014] Ducklow, H.W. (2003). Biogeochemical provinces: towards a JGOFS synthesis In Ocean Biogeochemistry (ed Fasham M. pp. 3–17). Springer, Berlin, Heidelberg, New York, pp. 3–17.

[ele13167-bib-0015] Edwards, K.F. , Thomas, M.K. , Klausmeier, C.A. & Litchman, E. (2012). Allometric scaling and taxonomic variation in nutrient utilization traits and maximum growth rate of phytoplankton. Limnol. Oceanogr., 57, 554–566.

[ele13167-bib-0016] Edwards, K.F. , Thomas, M.K. , Klausmeier, C.A. & Litchman, E. (2015). Light and growth in marine phytoplankton: allometric, taxonomic, and environmental variation. Limnol. Oceanogr., 60, 540–552.

[ele13167-bib-0017] Field, C.B. , Behrenfeld, M.J. , Randerson, J.T. & Falkowski, P. (1998). Primary production of the biosphere: integrating terrestrial and oceanic components. Science, 281, 237–240. 9657713 10.1126/science.281.5374.237

[ele13167-bib-0018] Finkel, Z.V. , Beardall, J. , Flynn, K.J. , Quigg, A. , Rees, T.A.V. & Raven, J.A. (2010). Phytoplankton in a changing world: cell size and elemental stoichiometry. J. Plankton Res., 32, 119–137.

[ele13167-bib-0019] Finlay, B.J. (2002). Global dispersal of free‐living microbial eukaryote species. Science, 296, 1061–1063. 12004115 10.1126/science.1070710

[ele13167-bib-0020] Fisher, R.A. (1930). Genetical Theory of Natural Selection. Claredon Press, Oxford.

[ele13167-bib-0021] Follows, M.J. & Dutkiewicz, S. (2011). Modeling diverse communities of marine microbes. Ann. Rev. Mar. Sci., 3, 427–451. 10.1146/annurev-marine-120709-142848 21329212

[ele13167-bib-0022] Fujiki, T. , Matsumoto, K. , Mino, Y. , Sasaoka, K. , Wakita, M. , Kawakami, H. *et al* (2014). Seasonal cycle of phytoplankton community structure and photo‐physiological state in the western subarctic gyre of the North Pacific. Limnol. Oceanogr., 59, 887–900.

[ele13167-bib-0023] Goebel, N.L. , Edwards, C.A. , Follows, M.J. & Zehr, J.P. (2014). Modeled diversity effects on microbial ecosystem functions of primary production, nutrient uptake, and remineralization. Ecology, 95, 153–163. 24649655 10.1890/13-0421.1

[ele13167-bib-0024] Grace, J.B. , Anderson, T.M. , Seabloom, E.W. , Borer, E.T. , Adler, P.B. , Harpole, W.S. *et al* (2016). Integrative modelling reveals mechanisms linking productivity and plant species richness. Nature, 529, 390–393. 26760203 10.1038/nature16524

[ele13167-bib-0025] Grover, J.P. (1990). Resource competition in a variable environment: phytoplankton growing according to Monod's model. Am. Nat., 136, 771–789.

[ele13167-bib-0026] Hector, A. , Schmid, B. , Beierkuhnlein, C. , Caldeira, M.C. , Diemer, M. , Dimitrakopoulos, P.G. *et al* (1999). Plant diversity and productivity experiments in European grasslands. Science, 286, 1123–1127. 10550043 10.1126/science.286.5442.1123

[ele13167-bib-0027] Hillebrand, H. & Matthiessen, B. (2009). Biodiversity in a complex world: consolidation and progress in functional biodiversity research. Ecol. Lett., 12, 1405–1419. 19849711 10.1111/j.1461-0248.2009.01388.x

[ele13167-bib-0028] Hooper, D.U. , Chapin, F.S. , Ewel, J.J. , Hector, A. , Inchausti, P. , Lavorel, S. *et al* (2005). Effects of biodiversity on ecosystem functioning: a consensus of current knowledge. Ecol. Monogr., 75, 3–35.

[ele13167-bib-0029] Hooper, D.U. , Adair, E.C. , Cardinale, B.J. , Byrnes, J.E.K. , Hungate, B.A. , Matulich, K.L. *et al* (2012). A global synthesis reveals biodiversity loss as a major driver of ecosystem change. Nature, 486, 105–108. 22678289 10.1038/nature11118

[ele13167-bib-0030] Huisman, J. (2010). Comment on ‘Patterns of diversity in marine phytoplankton’. Science, 329, 512–512. 10.1126/science.1189880 20671171

[ele13167-bib-0031] Huston, M.A. (1997). Hidden treatments in ecological experiments: re‐evaluating the ecosystem function of biodiversity. Oecologia, 110, 449–460. 28307235 10.1007/s004420050180

[ele13167-bib-0032] Hutchinson, G.E. (1961). The paradox of the plankton. Am. Nat., 95, 137–145.

[ele13167-bib-0033] Kashtan, N. , Roggensack, S.E. , Rodrigue, S. , Thompson, J.W. , Biller, S.J. , Coe, A. *et al* (2014). Single‐cell genomics reveals hundreds of coexisting subpopulations in wild Prochlorococcus. Science, 344, 416–420. 24763590 10.1126/science.1248575

[ele13167-bib-0034] Krause, S. , Le Roux, X. , Niklaus, P.A. , Van Bodegom, P.M. , Lennon, J.T. , Bertilsson, S. *et al* (2014). Trait‐based approaches for understanding microbial biodiversity and ecosystem functioning. Front. Microbiol., 5, 1–10. 10.3389/fmicb.2014.00251. 24904563 PMC4033906

[ele13167-bib-0035] Laws, E.A. , Falkowski, P.G. , Smith, W.O. , Ducklow, H. & McCarthy, J.J. (2000). Temperature effects on export production in the open ocean. Global Biogeochem. Cycles, 14, 1231–1246.

[ele13167-bib-0036] Litchman, E. & Klausmeier, C.A. (2008). Trait‐based community ecology of phytoplankton. Ann. Rev. Ecol. Evol. Syst., 39, 615–639.

[ele13167-bib-0037] Litchman, E. , Klausmeier, C.A. , Schofield, O.M. & Falkowski, P.G. (2007). The role of functional traits and trade‐offs in structuring phytoplankton communities: scaling from cellular to ecosystem level. Ecol. Lett., 10, 1170–1181. 17927770 10.1111/j.1461-0248.2007.01117.x

[ele13167-bib-0038] Loreau, M. (2010). From Populations to Ecosystems: Theoretical Foundations for a New Ecological Synthesis. Princeton University Press, Princeton, NJ.

[ele13167-bib-0039] Loreau, M. & Hector, A. (2001). Partitioning selection and complementarity in biodiversity experiments. Nature, 412, 72–76. 11452308 10.1038/35083573

[ele13167-bib-0040] Loreau, M. , Naeem, S. , Inchausti, P. , Bengtsson, J. , Grime, J.P. , Hector, A. *et al* (2001). Biodiversity and ecosystem functioning: current knowledge and future challenges. Science, 294, 804–808. 11679658 10.1126/science.1064088

[ele13167-bib-0041] Mandal, S. , Locke, C. , Tanaka, M. & Yamazaki, H. (2014). Observations and models of highly intermittent phytoplankton distributions. PLoS ONE, 9, e94797 10.1371/journal.pone.0094797. 24787740 PMC4008380

[ele13167-bib-0042] Marañón, E. (2015). Cell size as a key determinant of phytoplankton metabolism and community structure. Ann. Rev. Mar. Sci., 7, 241–264. 10.1146/annurev-marine-010814-015955 25062405

[ele13167-bib-0043] Marañón, E. , Cermeño, P. , Latasa, M. & Tadonléké, R.D. (2012). Temperature, resources, and phytoplankton size structure in the ocean. Limnol. Oceanogr., 57, 1266–1278.

[ele13167-bib-0044] Marañón, E. , Cermeño, P. , López‐Sandoval, D.C. , Rodríguez‐ Ramos, T. , Sobrino, C. , Huete‐Ortega, M. *et al* (2013). Unimodal size scaling of phytoplankton growth and the size dependence of nutrient uptake and use. Ecol. Lett., 16, 371–379. 23279624 10.1111/ele.12052

[ele13167-bib-0046] Merico, A. , Bruggeman, J. & Wirtz, K. (2009). A trait‐based approach for downscaling complexity in plankton ecosystem models. Ecol. Mod., 220, 3001–3010.

[ele13167-bib-0047] Merico, A. , Brandt, G. , Smith, S.L. & Oliver, M. (2014). Sustaining diversity in trait‐based models of phytoplankton communities. Front. Ecol. Evol., 2, 59 10.3389/fevo.2014.00059.

[ele13167-bib-0048] Moore, C.M. , Mills, M.M. , Arrigo, K.R. , Berman‐Frank, I. , Bopp, L. , Boyd, P.W. *et al* (2013). Processes and patterns of oceanic nutrient limitation. Nat. Geosci., 6, 701–710.

[ele13167-bib-0049] Morel, F.M. (1987). Kinetics of nutrient uptake and growth in phytoplankton. J. Phycol., 23, 137–150.

[ele13167-bib-0050] Norberg, J. , Swaney, D.P. , Dushoff, J. , Lin, J. , Casagrandi, R. & Levin, S.A. (2001). Phenotypic diversity and ecosystem functioning in changing environments: a theoretical framework. Proc. Natl Acad. Sci., 98, 11376–11381. 11535803 10.1073/pnas.171315998 PMC58737

[ele13167-bib-0051] Odate, T. (1996). Abundance and size composition of the summer phytoplankton communities in the western North Pacific Ocean, the Bering Sea, and the Gulf of Alaska. J. Oceanogr., 52, 335–351.

[ele13167-bib-0052] Savage, V.M. , Webb, C.T. & Norberg, J. (2007). A general multi‐trait‐based framework for studying the effects of biodiversity on ecosystem functioning. J. Theor. Biol., 247, 213–229. 17448502 10.1016/j.jtbi.2007.03.007 PMC2041898

[ele13167-bib-0053] Schwartz, M.W. , Brigham, C.A. , Hoeksema, J.D. , Lyons, K.G. , Mills, M.H. & Van Mantgem, P.J. (2000). Linking biodiversity to ecosystem function: implications for conservation ecology. Oecologia, 122, 297–305. 28308280 10.1007/s004420050035

[ele13167-bib-0054] Shchepetkin, A.F. & McWilliams, J.C. (2005). The regional oceanic modeling system (ROMS): a split‐explicit, free‐surface, topography‐following‐coordinate oceanic model. Ocean Mod., 9, 347–404.

[ele13167-bib-0055] Smith, S.L. , Vallina, S.M. & Merico, A. (2016). Phytoplankton size‐diversity mediates an emergent trade‐off in ecosystem functioning for rare versus frequent disturbances. Sci. Rep., 6, 34170 10.1038/srep34170. 27748359 PMC5066229

[ele13167-bib-0056] Tilman, D. , Knops, J. , Wedin, D. , Reich, P. , Ritchie, M. & Siemann, E. (1997). The influence of functional diversity and composition on ecosystem processes. Science, 277, 1300–1302.

[ele13167-bib-0057] Tilman, D. , Isbell, F. & Cowles, J.M. (2014). Biodiversity and ecosystem functioning. Ann. Rev. Ecol. Evol. Syst., 45, 471–493.

[ele13167-bib-0058] Vallina, S.M. , Ward, B.A. , Dutkiewicz, S. & Follows, M.J. (2014a). Maximal feeding with active prey‐switching: A kill‐the‐winner functional response and its effect on global diversity and biogeography. Prog. Oceanogr., 120, 93–109.

[ele13167-bib-0059] Vallina, S.M. , Follows, M.J. , Dutkiewicz, S. , Montoya, J.M. , Cermeno, P. & Loreau, M. (2014b). Global relationship between phytoplankton diversity and productivity in the ocean. Nat. Comm., 5, 4299 10.1038/ncomms5299. PMC4102128 24980772

[ele13167-bib-0060] Vallina, S.M. , Cermeno, P. , Dutkiewicz, S. , Loreau, M. & Montoya, J.M. (2017). Phytoplankton functional diversity increases ecosystem productivity and stability. Ecol. Mod., 361, 184–196.

[ele13167-bib-0061] Wirtz, K.W. (2000). Second order up‐scaling: theory and an exercise with a complex photosynthesis model. Ecol. Mod., 126, 59–71.

[ele13167-bib-0062] Wirtz, K.W. (2011). Non‐uniform scaling in phytoplankton growth rate due to intracellular light and CO2 decline. J. Plankton Res., 33, 1325–1341.

[ele13167-bib-0063] Wirtz, K.W. (2014). A biomechanical and optimality‐based derivation of prey‐size dependencies in planktonic prey selection and ingestion rates. Mar. Ecol. Prog. Ser., 507, 81–94.

[ele13167-bib-0064] Wirtz, K.W. & Eckhardt, B. (1996). Effective variables in ecosystem models with an application to phytoplankton succession. Ecol. Mod., 92, 33–53.

[ele13167-bib-0065] Yachi, S. & Loreau, M. (1999). Biodiversity and ecosystem productivity in a fluctuating environment: the insurance hypothesis. Proc. Natl Acad. Sci., 96, 1463–1468. 9990046 10.1073/pnas.96.4.1463 PMC15485

