# Supplementary material for: Effect of phytoplankton size diversity on primary productivity in the North Pacific: trait distributions under environmental variability
Source: Ecol Lett. 2018 Oct 17;22(1):56–66. doi: 10.1111/ele.13167 (PMC7380061; doi:10.1111/ele.13167)
Supplement: Supplementary file 1 [file ELE-22-56-s001.pdf]

Supporting Information for

Effect of phytoplankton size diversity on primary productivity in the North Pacific: trait distributions under environmental variability

Bingzhang Chen*, S. Lan Smith, Kai W. Wirtz

*To whom correspondence should be addressed. Email: bingzhang.chen@gmail.com

This PDF file contains:

Supporting Text

Table S1

Figs. S1 to S8

References

Supporting Information

Contents

S.1 Supporting Text

S.1.1 Description of the three-dimensional (3D) regional ocean model

S.1.2 Description of the ecosystem model

S.1.3 Comparisons between model outputs and observational data

S.1.4 Modeled spatial patterns of phytoplankton properties

S.1.5 Temporal changes of phytoplankton size and productivity in two selected hotspots

S.1.6 Spatial patterns of covariances and second partial derivatives in surface waters

S.1.7 Idealized numerical experiments

S.2 Supporting Table

S.3 Supporting Figures

S.4 Supporting References

S.1 Supporting Text

S.1.1 Description of the three-dimensional (3D) regional ocean model

A regional 3D ocean model built on the Regional Ocean Modelling System (ROMS) (Shchepetkin & McWilliams 2005) was developed for the North Pacific (95.63 ºE to 285.43 ºE, 10.43 ºS to 66.57 ºN). ROMS is a terrain following, free surface ocean model that solves hydrostatic, primitive equations. The spatial resolution is 1° x 1° with 40 vertical layers having finer vertical resolution at the sea surface (surface and bottom stretching parameters being 2.0 and 0.6, respectively). The normal year datasets of coordinated ocean-ice reference experiments (CORE) version 2 containing heat and freshwater fluxes, and wind speeds were used as surface forcing (Large & Yeager 2009). Surface temperature and salinity data were relaxed to the climatology of the World Ocean Atlas (WOA) 2003 (version 2; Boyer et al. 2013).

The model was started assuming no water motion, with initial conditions (January 1st) of temperature and salinity interpolated from the WOA2013 climatology. It was integrated for ten years to achieve quasi steady state, and the results of final year were examined.

S.1.2 Description of the ecosystem model

The ecosystem model contains 8 tracers in total: dissolved inorganic nitrogen (DIN, abbreviated as $\boldsymbol{N}$ in all the equations; unit: μmol N L^{-1}), phytoplankton biomass ($\boldsymbol{P}$; μmol N L^{-1}), zooplankton biomass ($\boldsymbol{Z}$;

μmol N L^{-1}), detritus in terms of nitrogen (***D***; μmol N L^{-1}) and iron (***D***$_{\boldsymbol{Fe}}$; nmol Fe L^{-1}), dissolved iron (***fer***; nmol Fe L^{-1}), the products of $\boldsymbol{P\bar{l}}$ (abbreviated as $\boldsymbol{P}_l$) and $\boldsymbol{P(v+\bar{l}^2)}$ (abbreviated as $\boldsymbol{P}_v$) where $\boldsymbol{\bar{l}}$ (ln μm^3) is the phytoplankton mean log cell volume and $\boldsymbol{v}$ ((ln μm^3)^2) is the log volume variance (Fig. S1).

The equation for phytoplankton biomass is:

$$\frac{dP}{dt} = P\mu_{com} - f(E_z, T)G(P)Z + Adv(P) + Diff(P) \tag{1}$$

where μ_{com} is the phytoplankton specific growth rate (d^{-1}) of the whole community (i.e. integrated over the whole size spectra). The operators *Adv*() and *Diff*() describe three dimensional water advection and diffusion, respectively. The Arrhenius function $f(E,T) = e^{\frac{E_Z}{k}\left(\frac{1}{T_0}-\frac{1}{T}\right)}$ describes the temperature effects on rates with the activation energy E (in electron volts [eV], 1 eV = 96.49 kJ mol^{-1}). E_z and E_p denote for zooplankton and phytoplankton, respectively. The grazing function $G(P) = I_{max}\frac{P^2}{P^2+K_P^2}$ describes a Holling type III functional response of zooplankton grazing. I_{max} is the maximal per capita ingestion rate (d^{-1}), and K_P the half-saturation constant of grazing.

Following Merico *et al.* (2014) and Smith *et al.* (2016), the equations for μ_{com}, l, and v are:

$$\mu_{com} = \left(\mu + \frac{v}{2}\left(\frac{d^2\mu}{dl^2} + u\frac{d^4\mu}{dl^4}\right) - 3u\frac{d^3\mu}{dl^3}\right)\Big|_{l=\bar{l}} \tag{2}$$

$$\frac{d\bar{l}}{dt} = \left[v\left(\frac{d\mu}{dl} + u\frac{d^3\mu}{dl^3}\right) - 3u\frac{d\mu}{dl}\right]\Big|_{l=\bar{l}} \tag{3}$$

$$\frac{dv}{dt} = \left\{v\left[v\left(\frac{d^2\mu}{dl^2} - \frac{d^2g}{dl^2} + u\frac{d^4\mu}{dl^4}\right) - 5u\frac{d^2\mu}{dl^2}\right] + 2u\mu\right\}\Big|_{l=\bar{l}} \tag{4}$$

where μ is the phytoplankton growth rate (d^{-1}) at mean size $\bar{l}$. u is the trait diffusion parameter. $\frac{d^2g}{dl^2}$ is the second derivative of zooplankton clearance rate (d^{-1}) against phytoplankton size and can be derived from the log-normal prey size distribution underlying our moment approximation and a log-normal size size grazing kernel consistent with empirical data and mechanistic considerations (Wirtz 2014). This grazing kernel $e^{-s(l-l_{opt})^2}$ introduces the selectivity s as the inverse feeding breadth. The KTW mechanism implies that the optimal prey size (l_{opt}) of zooplankton grazing always coincides with the mean (log) size of the phytoplankton size distribution.

The second order derivative of the specific rate then fulfils the condition (Smith *et al.* 2016):

$$\frac{d^2g}{dl^2} = -\frac{\alpha_g}{v} g(l)\Big|_{l=\bar{l}} \tag{5}$$

in which

$$g(l)|_{l=\bar{l}} = \frac{G(P)}{P}\sqrt{1+\alpha_g} \tag{6}$$

$\alpha_g > 0$ means zooplankton is engaged in selective grazing and has a feeding preference on abundant prey.

The equations of $\boldsymbol{P_l}$ and $\boldsymbol{P_v}$ follow:

$$\frac{dP_l}{dt} = P\frac{d\bar{l}}{dt} + \bar{l}\frac{dP}{dt} + Adv(P_l) + Diff(P_l) \tag{7}$$

$$\frac{dP_v}{dt} = P\left(\frac{dv}{dt} + 2\bar{l}\frac{d\bar{l}}{dt}\right) + \left(v + \bar{l}^2\right)\frac{dP}{dt} + Adv(P_v) + Diff(P_v) \tag{8}$$

The total ingested food by zooplankton is divided into net growth, excretion into the inorganic nitrogen pool, and defecation of unassimilated food into the detritus pool (Buitenhuis *et al.* 2010). Zooplankton mortality is set to be proportional to the squares of its biomass and is also converted into detritus pool. As such, the dynamics of zooplankton follow:

$$\frac{dZ}{dt} = f(E_z, T)(ZG(P)NGE - m_z Z^2) + Adv(Z) + Diff(Z) \qquad (9)$$

where *NGE* is the net growth efficiency of zooplankton. m_z is the mesozooplankton mortality coefficient (d^{-1} (μmol N $L^{-1})^{-1}$).

Detritus is converted to DIN at a rate (R_{dn}, d^{-1}) that has the same temperature sensitivity with zooplankton grazing. Detritus is also assumed to have a sinking rate (W_d, d^{-1}) that linearly relates to surface temperature (*SST*) (Gregg 2008).

$$W_d = W_0(k + bSST) \qquad (10)$$

$$\frac{dD}{dt} = f(E_z, T)(ZG(P)unass + m_z Z^2 - DR_{dn}) - W_d \frac{dD}{dz} + Adv(D) + Diff(D) \qquad (11)$$

where *unass* represents the fraction of unassimilated food by zooplankton.

DIN is taken up by phytoplankton and is replenished by zooplankton excretion and detritus regeneration.

$$\frac{dN}{dt} = -P\mu_{com} + f(E_z, T)(ZG(P)(1 - NGE - unass) + DR_{dn}) + Adv(N) + Diff(N) \qquad (12)$$

The sources and sinks of *fer* largely follow DIN with an additional source (atmospheric deposition; Fe_{depo}) and sink (scavenging; fer_{scav}) (Nickelsen *et al.* 2015):

$$\frac{dfer}{dt} = \begin{bmatrix} -P\mu_{com} + f(E_z, T)(ZG(P)(1 - NGE - unass) \\ +DR_{dn}) \end{bmatrix} R_{fer\_N} + Fe_{depo} - fer_{scav} + Adv(fer) + Diff(fer) \quad (13)$$

To translate between nitrogen and iron in phytoplankton and zooplankton, a constant *fer*:*N* ratio (R_{fer_N}) of 0.0265 is assumed. The data of monthly atmospheric deposition of total soluble iron are extracted from the Scenario III in Luo *et al.* (2008). Iron scavenging rate (fer_{scav}) is composed of both linear scavenging rate (k_{scm}, d^{-1}) and particle absorption rate (k_{sc}, d^{-1} (μM N)$^{-1}$) (Nikelsen *et al.* 2015):

$$fer_{scav} = (k_{scm} + k_{sc}Df(E_z, T))Fe_{prime} \quad (14)$$

in which Fe_{prime} is the concentration (nmol L^{-1}) of free iron:

$$Fe_{prime} = \frac{\left(-A + \sqrt{4ferk_{eq} + A^2}\right)}{2k_{eq}} \quad (15)$$

$$A = 1 + \left(l_{fe} - fer\right)k_{eq} \quad (16)$$

where k_{eq} (unit: (mol lig L^{-1})$^{-1}$) is the equilibrium constant between free iron and ligands and is assumed to depend only on temperature:

$$k_{eq} = 10^{\left(17.27 - \frac{1565.7}{T}\right)} \quad (17)$$

Note that T is in absolute temperature (K). l_{fe} is the total iron ligand concentration that is assumed constant (0.6 nmol lig L^{-1}).

The equation for $\boldsymbol{D_{Fe}}$ is:

$$\frac{dD_{Fe}}{dt} = f(E_z, T)(ZG(P)unass + m_z Z^2 - R_{dn}D)R_{fe\_N} - W_d\frac{dD_{Fe}}{dz} + fer_{scav} + Adv(D_{Fe}) + Diff(D_{Fe}) \quad (18)$$

Phytoplankton growth rate (μ) depends on temperature (T, K), light (I, W m^{-2}), DIN and *fer*:

$$\mu = f(E_p, T)\mu_m \min\left(\frac{N}{N+K_N}, \frac{fer}{fer+K_{fer}}\right)\left(1 - e^{-\frac{\alpha_C I}{\mu_0 f(E_p,T)}}\right) \quad (19)$$

The trait parameters μ_m, K_N, $K_{fer,}$ and α_c are all dependent on cell size ***l***:

$$\mu_m = \mu_0 e^{\alpha_\mu l + \beta_\mu l^2} \quad (21)$$

$$K_N = K_{0,N} e^{\alpha_K l} \quad (22)$$

$$K_{fer} = K_{0,fer} e^{\alpha_{fer} l} \quad (23)$$

$$\alpha_c = \alpha_{0,c} e^{\alpha_I l} \quad (24)$$

Phytoplankton chlorophyll-to-carbon (θ, g Chl (mol C$)^{-1}$) and nitrogen-to-carbon (Q_N, mol N (mol C$)^{-1}$) ratios are calculated from ambient light and nutrient levels:

$$\theta = \theta_{min} + \frac{\mu}{I\alpha_C}(\theta_{max} - \theta_{min}) \quad (25)$$

$$Q_N = \frac{Q_{min}}{1 - \left(1 - \frac{Q_{min}}{Q_{max}}\right) min\left(\frac{N}{N+K_N}, \frac{fer}{fer+K_{fer}}\right)} \quad (26)$$

where θ_{min} and θ_{max} are minimal and maximal Chl:C ratios, respectively. Q_{min} and Q_{max} are minimal and maximal N:C ratios, respectively. Total Chl *a* concentrations (*Chl*, μg L^{-1}) and net primary production (*NPP*, μgC L^{-1} d^{-1}) integrated over the whole size range can be calculated as:

$$Chl = P\left(\frac{\theta}{Q_N} + \frac{v}{2}\frac{d^2\left(\frac{\theta}{Q_N}\right)}{dl^2}\right)\Bigg|_{l=\bar{l}} \quad (27)$$

$$NPP = P\left(\frac{\mu}{Q_N} + \frac{v}{2}\frac{d^2\left(\frac{\mu}{Q_N}\right)}{dl^2}\right)\Bigg|_{l=\bar{l}} \quad (28)$$

Light levels (I_z) at depth z were calculated based on PAR_0 and Chl *a* concentrations following the Beer-Lambert law:

$$I_z = PAR_0 e^{-\left(zK_w + K_{chl}\int_0^z Chl(x)dx\right)} \quad (29)$$

in which K_w and K_{chl} are the attenuation coefficients for seawater and Chl *a*, respectively. To realistically estimate the average light field that a phytoplankton cell should experience in the surface mixed layer (Franks 2015), the ambient light level for phytoplankton is calculated as the average light throughout this layer. All the parameter descriptions and values are shown in Table S1.

The initial conditions (January 1st) of inorganic nitrogen were interpolated from the WOA2013. The initial conditions of phytoplankton biomass were estimated from the SeaWIFS chlorophyll monthly climatology with the chlorophyll-to-carbon and nitrogen-to-carbon ratios calculated from the ambient nitrate, light, and temperature following the model developed by Pahlow *et al.* (2013). The vertical

profiles of chlorophyll were calculated based on surface values following Morel and Berthon (1989). The initial conditions of zooplankton and detritus were assumed 0.78 and 0.2 times the phytoplankton biomass, respectively, following previous model results (Ward *et al.* 2012). Initial $\bar{\boldsymbol{l}}$ and $\boldsymbol{v}$ were set globally set to 1 log μm^3 (approximating an equivalent spherical diameter of 1.7 μm) and 1 $(\log \mu m^3)^2$, respectively. The initial *Fer* conditions were extracted from the output of the Pelagic Interaction Scheme for Carbon and Ecosystem Studies (PISCES) ocean biogeochemical model that has been validated against iron fertilization experiments (Aumont & Bopp 2006). *DETFe* was initialized as 10^{-3} (mol Fe (mol N)$^{-1}$) of phytoplankton biomass.

We admit several limitations of our model. For example, N_2 fixation is not taken into account. We already know that in terrestrial BEF experiments, the complementarity between N_2 fixing legumes and other plants is an important factor leading to the positive BEF relationship. While N_2 fixation is certainly important in some parts of the North Pacific, the active process of denitrification due to the low oxygen environment essentially maintains the subtropical North Pacific limited by nitrogen. This suggests that we need to incorporate denitrification as well as oxygen cycle into the model if we plan to include N_2 fixation, which will add more complexity and uncertainty. Another limitation is that although we included two different nutrients, dissolved nitrogen and iron, phytoplankton species with different sizes

do not differ in their uptake ratios of N: Fe according to the common parameterizations of size dependency of nutrient uptake on nitrogen and iron (Ward *et al.* 2012). Thus, we are essentially simulating a phytoplankton community competing for a single set of nutrients, which however does not imply that different size classes cannot coexist.

S.1.3 Comparisons between model outputs and observational data

We compared model outputs of dissolved inorganic nitrogen (DIN, μM), Chl *a* concentrations (μg L^{-1}), net primary production (NPP, μg C L^{-1} d^{-1}) and fractions of picophytoplankton (<2 μm) from the simulation using the largest "trait diffusion" (TD) coefficient with observational data (Fig. S2). The outputs from the simulation using the largest "kill-the-winner" (KTW) coefficient are largely similar with those using the largest TD coefficient. Reducing the coefficients of TD or KTW does not have a large impact on bulk properties such as nutrient, Chl *a*, or NPP, but has some effects on size structure (*see* Fig. 2 of the main text).

We used the nitrate data of World Ocean Atlas (WOA) 2013 (version 2; Garcia *et al.* 2014) as the observational data for DIN. Chl *a* of SeaWIFS satellite monthly climatology product (https://oceancolor.gsfc.nasa.gov/SeaWiFS/) was used to compare with model outputs. For NPP, two

versions (vgpm and cbpm) of NPP data were downloaded from https://www.science.oregonstate.edu/ocean.productivity, which give different patterns of NPP. The vgpm and cbpm versions of NPP data were calculated based on the algorithms in Behrenfeld & Falkowski (1997) and Westberry *et al.* (2008), respectively. We used the algorithm (equation 2) in Ward (2015) to calculate the fractions of picophytoplankton from observed total Chl *a* and temperature.

The model largely captured the high levels of nitrate and Chl *a* in the subarctic and equatorial Pacific, which contrasted with the low nitrate and Chl *a* in the subtropics. Although Chl *a* may be overestimated in the subarctic and equatorial regions, satellite observations may underestimate Chl *a* in winter (data not shown). As such, the modeled fractions of picophytoplankton were lower than those predicted based on the algorithm in Ward (2015) since lower fractions of picophytoplankton were associated with higher Chl concentrations. The two algorithms, *vgpm* and *cbpm*, gave different patterns of NPP. The algorithm of *vgpm* predicted higher NPP in the subarctic, while *cbpm* predicted higher NPP in the equatorial region. The model patterns were between the two empirical algorithms, predicting the highest NPP in the equatorial region and moderately high NPP in the subarctic. The Taylor diagram shown in Fig. S2 suggests general correspondence between observations and model outputs, particularly for nitrate, Chl, and fractions of picophytoplankton.

We also compared model outputs with the data of nitrate and Chl *a* concentrations collected within the surface mixed layer at five times-series stations (ALOHA (158 °W, 22.75 °N), S1 (145 °E, 30 °N), K2 (160 °E, 47 °N), P (145 °W, 50 °N), Equatorial Pacific (140 °W, 0 °N); Fig. S3). The data of station ALOHA were downloaded from http://hahana.soest.hawaii.edu/hot/. The data at stations K2 and S1 were obtained from the K2S1 project (https://ebcrpa.jamstec.go.jp/k2s1/en/index.html). The data of three other stations were obtained from the JGOFS dataset (https://rda.ucar.edu/datasets/ds259.0/). While the model underestimated (e.g. ALOHA) or overestimated nitrate and Chl *a* concentrations (e.g. S1), it roughly captures the main seasonal cycles and spatial differences across a large environmental gradient in the North Pacific.

S.1.4 Spatial patterns of phytoplankton properties

Simulated patterns of important ecosystem variables are displayed in Fig. S4. Dissolved iron concentrations were highest at middle latitudes (20~40 °N), consistent with the high rate of atmospheric dust deposition there. Similar to Chl *a* concentration, phytoplankton biomass was also higher in the subarctic and equatorial regions, but lower in the subtropics. Based on the patterns of phytoplankton growth rates, mean size, the extents of light, nitrogen and iron limitation, N:C and Chl:C ratios, the

North Pacific can be classified into three main provinces: subarctic, subtropical and eastern equatorial Pacific.

The subarctic North Pacific is characterized by low temperature, relatively low light, high nitrate, large phytoplankton size and high biomass. Although the atmospheric dust deposition is not low in the subarctic Pacific, abundance in nitrate increases iron limitation of phytoplankton growth. But in general, light makes the main limitation factor in the subarctic and Chl:C and N:C ratios are generally high.

The subtropical Pacific the most oligotrophic area in the whole North Pacific, characterized by low nitrogen concentration due to strong water column stratification. The phytoplankton community is dominated by picophytoplankton. Light levels usually allow for sufficient productivity, also due to a shallow mixed layer. Due to the dual effects of strong light and low nitrogen, Chl:C and N:C ratios are low.

The eastern equatorial Pacific is characterized by strong upwelling, which leads to deep mixing and transport of nitrate into surface waters. This also leads to the paradoxical pattern that although surface light is strong in the equatorial regions, phytoplankton can be light-limited due to deep mixing. Also due to the scarce dust deposition, iron instead of nitrogen generally limits phytoplankton growth and particularly phytoplankton size. Therefore, the eastern equatorial Pacific is similar to the subarctic in

many aspects except for temperature. Consequently, the highest primary production is found in this area, consistent with the output of the *cbpm* algorithm.

S.1.5 Temporal changes of phytoplankton size and productivity in two selected hotspots

To more thoroughly understand the effects of size diversity on productivity, we select two hotspots where the effects of size diversity on productivity were the most positive (hotspot A) and negative (hotspot B), respectively (Fig. S5). The hotspot A was located within the North Pacific subtropical gyre, while the hotspot B was in the eastern equatorial Pacific.

At hotspot A, the $\mu|_{l=\bar{l}}$ (i.e. the growth rate of the dominant species) in the high diversity treatment was usually higher than those in the low diversity treatment, suggesting that the size of the dominant species was closer to the optimal size in the highest than in the lowest diversity treatment (Fig. S6). This behavior is inherent to our model equations and is consistent with the insurance hypothesis that the high diversity confers the phytoplankton community to more rapidly adapt to the changing environment by maintaining more species (Yachi & Loreau 1999; Norberg *et al.* 2001; Merico *et al.* 2014; Smith *et al.* 2016; Vallina *et al.* 2017). One side effect of this was that the higher μ was often associated with a more negative $\frac{d^2\mu}{dl^2}$ (i.e. the difference of growth rates between productive and unproductive

species is greater when growth rates are higher), because higher growth tends to induce stronger interspecific competition (Hutson 1979). In addition, the community becomes more diverse, containing more unproductive species (i.e. larger *v*), and thereby reducing the productivity at the community level. In spite of the negative impacts of larger *v* and more negative $\frac{d^2\mu}{dl^2}$, the community productivity calculated as $\left(\mu + \frac{v}{2}\frac{d^2\mu}{dl^2}\right)\Big|_{l=\bar{l}}$ was still larger in the high diversity treatment, although the differences were small (Fig. S6g).

At hotspot B, despite of the differences in the temporal trajectories of mean sizes due to the different size diversity, growth rates were almost identical between the two treatments (Fig. S6j-l). Due to the higher size diversity and negative $\frac{d^2\mu}{dl^2}$ values, the high diversity treatment had the slightly lower community productivity than the low diversity treatment, but the differences were negligible (Fig. S6n).

The key difference between the oligotrophic hotspot A and mesotrophic hotspot B is that in A, the sensitivity of μ to mean size $\bar{l}$ was greater than in B so that faster adjusting $\bar{l}$ gave greater increases in growth rate μ. These adjustments reflect a model community that has to optimize the tradeoff between achieving the highest productivity by retaining less unproductive species and the capacity to adapt to environmental fluctuation by increasing diversity. In general, the adaptive capacity is preferred when environmental fluctuation amplitudes are large (Smith *et al.* 2016).

S.1.6 Spatial patterns of covariances and second partial derivatives in surface waters

In Fig. S7, we compare the spatial distributions of temporal variances of $\bar{l}$ ($\sigma_{\bar{l}}^2$), covariances between $\bar{l}$ and light (σ_{lI}), nitrate (σ_{lN}), and iron (σ_{lfer}) in surface waters in both low- and high-diversity treatments. Compared to the low diversity treatment, $\sigma_{\bar{l}}^2$ increased substantially along the fronts between the central gyre and the two adjoining subarctic and equatorial Pacific. Note that because $\left.\frac{\partial^2\mu}{\partial\bar{l}^2}\right|_{\bar{l}=\bar{l}_0}$ was usually negative, increases of temporal variances of mean size in the high diversity treatment actually reduced phytoplankton growth rate. The enhancement of σ_{lfer} in the high diversity treatment was evident only in a few coastal areas. $\sigma_{\bar{l}N}$ also increased substantially along the fronts in the high-diversity treatment and also decreased evidently in the subarctic Pacific where phytoplankton growth is mostly limited by light. Comparatively, the enhancement of $\sigma_{\bar{l}N}$ was mostly pronounced in the light-limiting subarctic Pacific, while it mostly decreased in the western Pacific east of Japan.

S.1.7 Idealized numerical experiments

In the continuous case of the idealized numerical experiments, the model resolves only two compartments, nutrient (*N*) and phytoplankton (*P*).

$$\frac{dN}{dt} = N_0(t) - DN - P\mu_{com} \tag{30}$$

$$\frac{dP}{dt} = P(\mu_{com} - m) \tag{31}$$

where D is the nutrient turnover rate (d^{-1}). μ_{com} is the phytoplankton community-based growth rate (d^{-1}).

As in the 3D simulations, we assume that phytoplankton size (log cell volume l, ln μm^3) follow a normal distribution with the mean $\bar{l}$ and variance v ((ln $\mu m^3)^2$). The average growth rate of the community can then be calculated as:

$$\mu_{com} = \left(\mu + \frac{v}{2}\frac{d^2\mu}{dl^2}\right)\Big|_{l=\bar{l}} \tag{31}$$

where $\mu(\bar{l})$ is the growth rate at mean size, which depends on nutrient concentration N.

$$\mu(l) = \mu_m(l)\frac{N}{N+K_N(l)} \tag{32}$$

in which both μ_m and K_N are functions of l, similar to the 3D simulations:

$$\mu_m(l) = \mu_{0,m}e^{\alpha_\mu l + \beta_\mu l^2} \tag{33}$$

$$K_N(l) = K_{0,N}e^{\alpha_K l} \tag{34}$$

Nutrient inflow $N_0(t)$ is a seasonally varying sinusoidal function:

$$N_0(t) = S_0\left(1 + A\sin\frac{2\pi t}{\lambda}\right) \tag{35}$$

where S_0 is the time-averaged nutrient supply. A and λ are the amplitude and period of the nutrient fluctuation, respectively.

In the simulations of investigating relationships between primary production and species richness (R), we randomly sample R species ($1 \leq R \leq 10$) with the sizes between 0.5 μm and 200 μm. The dynamics of nutrients and phytoplankton follow:

$$\frac{dN}{dt} = N_0(t) - DN - \sum_{i=1}^{R} P_i \mu_i \quad (36)$$

$$\frac{dP_i}{dt} = P_i(\mu_i - m) \quad (37)$$

where P_i and μ_i are the biomass and growth rate of the ith species. μ_i depends on the size of the species as in Eq. (32-34).

The following parameters were used in the simulation:

$S_0 = 0.3$ μM N d^{-1}; $A = 0.4$ and 0.8 d^{-1}; $\lambda = 360$ d; $D = 0.3$ d^{-1}; $m = 0.5$ d^{-1}; $\mu_{0,m} = 0.8$ d^{-1}; $a_{\mu} = 0.2$ (ln μm^3)$^{-1}$; $\beta_{\mu} = -0.01$ (ln μm^3)$^{-2}$; $K_{0,N} = 0.2$ μM N; $a_K = 0.27$ (ln μm^3)$^{-1}$.

The initial N and total P are 1 and 0.001 μM N, respectively.

S.2 Supporting Table S1. Parameters of the ecosystem model with a continuous size distribution for phytoplankton.

Symbol	Description	Value	Unit
K_w	Light attenuation coefficient of seawater	0.04[a]	m^{-1}
K_{chl}	Light attenuation coefficient of chlorophyll	0.025[a]	(mg Chl *a* m^{-2}) $^{-1}$
E_p	Activation energy of phytoplankton rates	0.41[b]	eV
E_z	Activation energy of heterotrophic rates	0.65[b]	eV
θ_{min}	Minimal chlorophyll-to-carbon ratio	0.02[c]	gChl $molC^{-1}$
θ_{max}	Maximal chlorophyll-to-carbon ratio	0.62[d]	gChl $molC^{-1}$
unass	Fraction of unassimilated food by zooplankton	0.24[e]	dimensionless
NGE	Net growth efficiency of zooplankton	0.3[e]	dimensionless
R_{dn}	Conversion rate of detritus to inorganic nitrogen	0.1[f]	d^{-1}
l_{fe}	Total iron ligand concentration	0.6 [f]	nM
K_{scm}	Minimal iron scavenging rate	5×10^{-3} [f]	d^{-1}
K_{sc}	Particle dependent scavenging rate	0.03[f]	(μM N)$^{-1}$ d^{-1}
R_{Fe_N}	Plankton iron-to-nitrogen ratio	0.0265	nM:μM
$\mu'_{0,m}$	Phytoplankton maximal growth rate at 1 μm^3 at 15 °C	0.8[b]	d^{-1}
a_μ	First-order size scaling component for μ_m	0.2 [b]	(ln μm^3)$^{-1}$
β_μ	Second-order size scaling component for μ_m	-0.01[b]	(ln μm^3)$^{-2}$
a_K	Size scaling exponent for K_N	0.27[g]	(ln μm^3)$^{-1}$
a_{fer}	Size scaling exponent for K_{fer}	0.27[g]	(ln μm^3)$^{-1}$
Q_{min}	Phytoplankton minimal N:C ratio	0.06[h]	mol N: mol C
Q_{max}	Phytoplankton maximal N:C ratio	0.18[h]	mol N: mol C
$a_{0,c}$	Initial slope of photosynthesis versus light at 1 μm^3	0.03[i]	(W m^{-2})$^{-1}$ d^{-1}
a_I	Size scaling exponent for a_c	–0.13[i]	(ln μm^3)$^{-1}$
W_0	Normalized sinking rate of detritus	20[j]	m d^{-1}
k	Intercept of detrital sinking rate vs. SST	0.3[j]	dimensionless

b	Slope of detrital sinking rate vs. SST	0.03[j]	ºC^{-1}
g_m	Maximal microzooplankton ingestion rate at 15 ºC	1.0[k]	d^{-1}
m_z	Coefficient of zooplankton mortality at 15 ºC	0.1[a]	(μM N)$^{-1}$ d^{-1}
K_p	Grazing half-saturation constant of zooplankton	0.5[k]	μM N
$K_{0,N}$	Growth half-saturation constant for nitrogen for a phytoplankton cell of 1 μm^3	0.2[g]	μM N
$K_{0,fer}$	Growth half-saturation constant for iron of phytoplankton with 1 μm^3	0.02[g]	nM Fe

[a]Fennel *et al.* (2006); [b]Chen & Laws (2017); [c]Flynn (2003); [d]Pahlow *et al.* (2013); [e]Buitenhuis *et al.* (2010); [f]Nickelsen *et al.* (2015); [g]Ward *et al.* (2012); [h]Marañón *et al.* (2013); [i]Edwards *et al.* (2015); [j]Gregg (2008); [k]Chai et al. (2002).

S.3 Supporting Figures

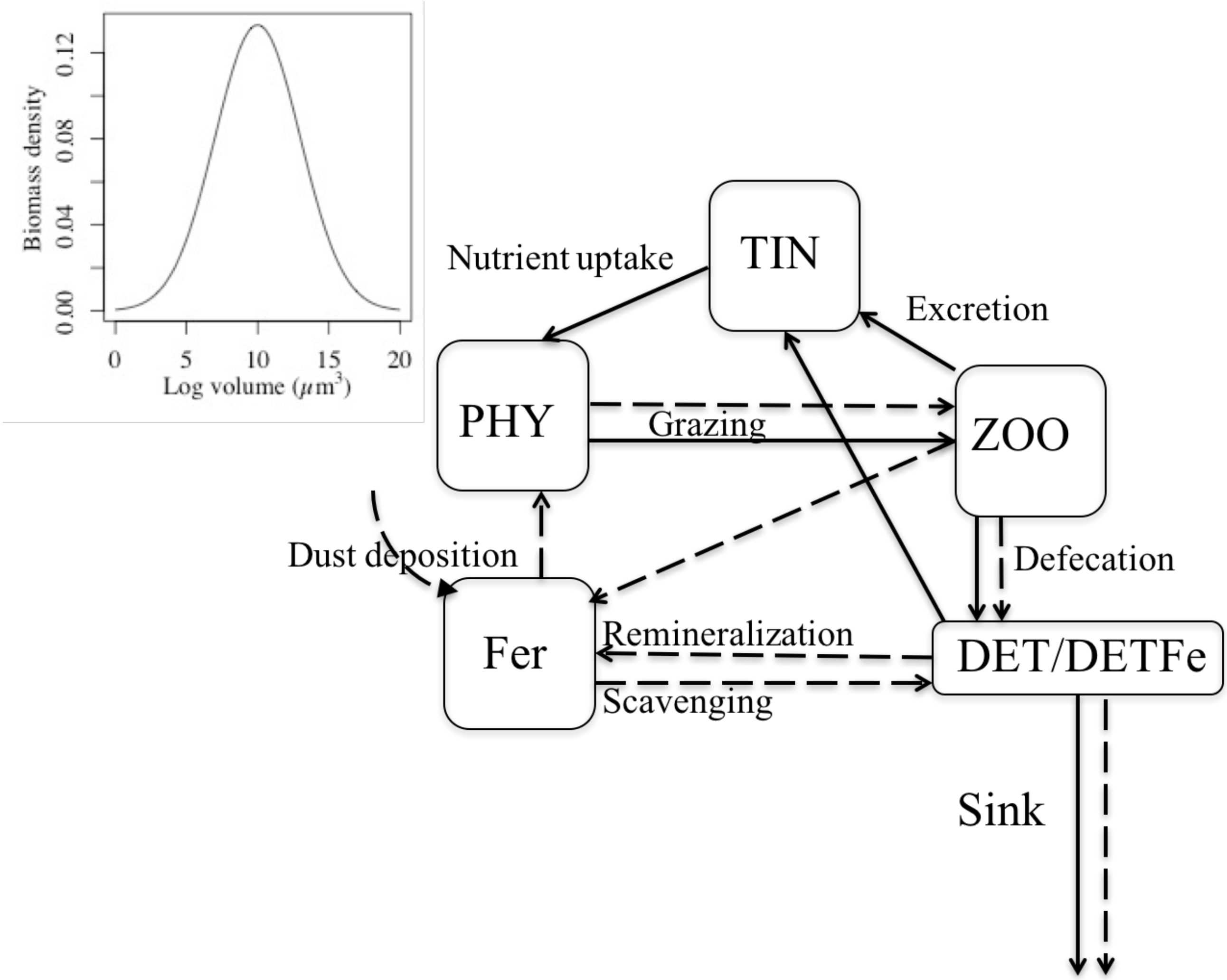

Fig. S1 Schematic description of the ecosystem model with the continuous size distribution for phytoplankton. Thick arrows indicate nitrogen flows and dashed lines indicate the iron cycle. The inset denotes a phytoplankton community with a lognormal size distribution.

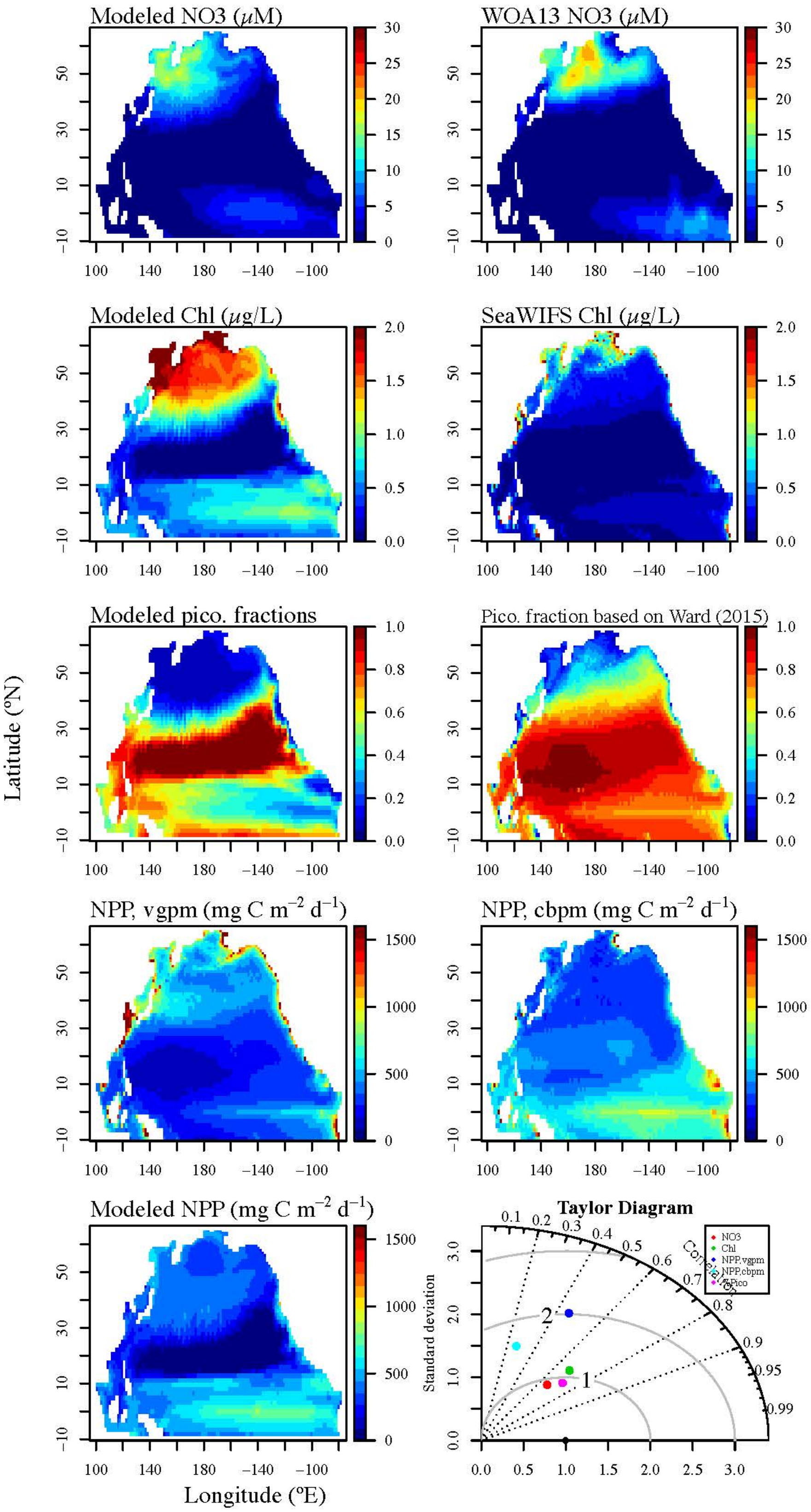
Modeled NO3 (μM)
WOA13 NO3 (μM)
Modeled Chl (μg/L)
SeaWIFS Chl (μg/L)
Modeled pico. fractions
Pico. fraction based on Ward (2015)
NPP, vgpm (mg C m^{-2} d^{-1})
NPP, cbpm (mg C m^{-2} d^{-1})
Modeled NPP (mg C m^{-2} d^{-1})
Taylor Diagram
Latitude (ºN)
Longitude (ºE)
Standard deviation
Correlation
NO3
Chl
NPP,vgpm
NPP,cbpm
Pico
100
140
180
−140
−100
−10
10
30
50

Fig. S2 Comparison of annual spatial patterns of nitrate, Chl *a* concentration, net primary production (NPP, μg C L^{-1} d^{-1}), and fraction of picophytoplankton (<2 μm) between the simulation using the largest "trait diffusion" (TD) coefficient and observational data. The right panel at bottom is a Taylor diagram that summarizes the comparisons between model and data. The correlation coefficients between the model and data are indicated by the values (0.1~0.99) along the arc. The distances between the origin and the color points indicate the normalized standard deviation. The distances between the reference point [1, 0] and the color points indicate the root mean squared error.

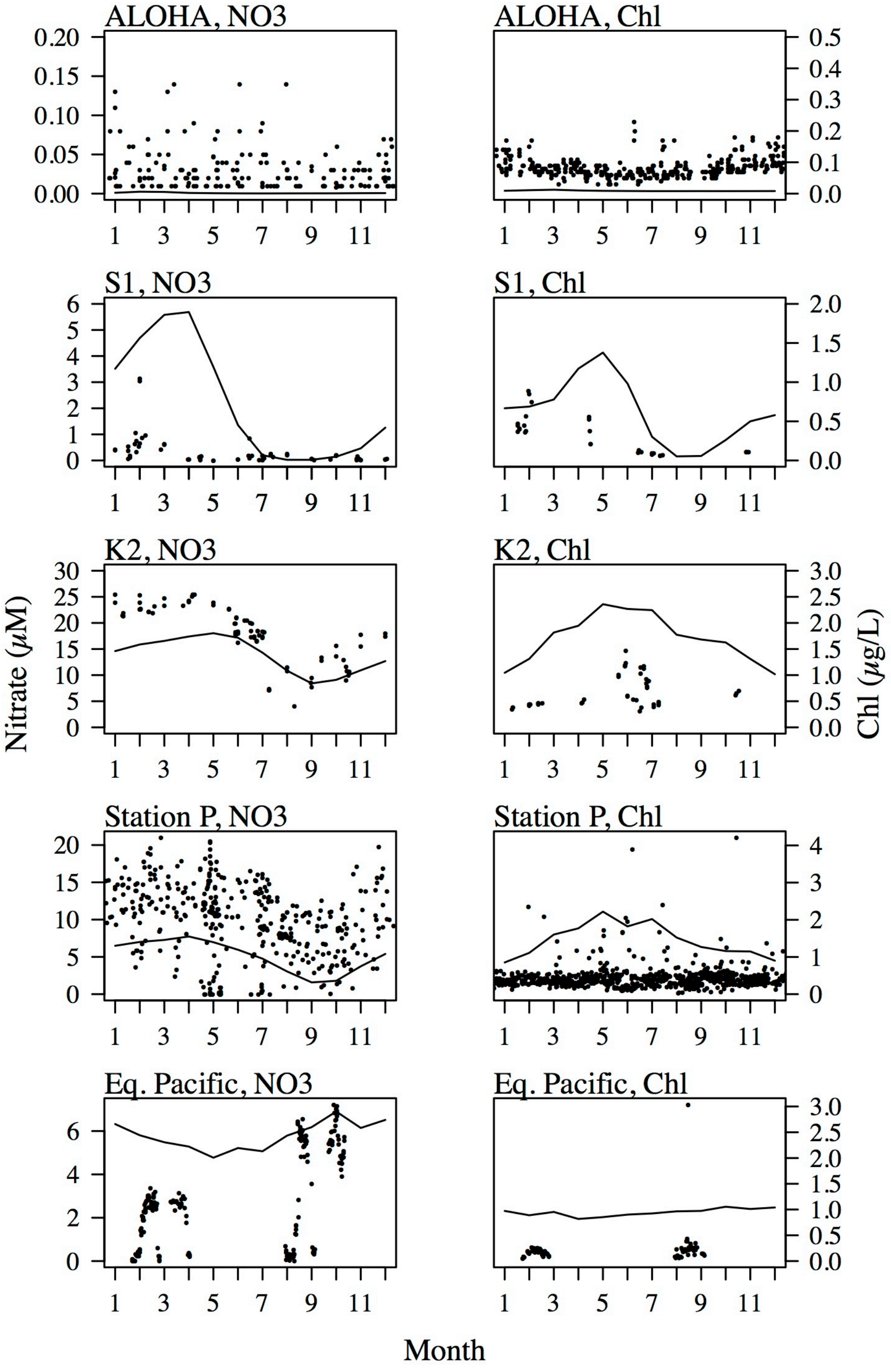

Fig. S3 Comparisons of seasonal cycles of nitrate (left column) and Chl *a* concentrations (right column) within the surface mixed layer at five time-series observational stations in the North Pacific. Solid lines represent model outputs and dots represent observational data.

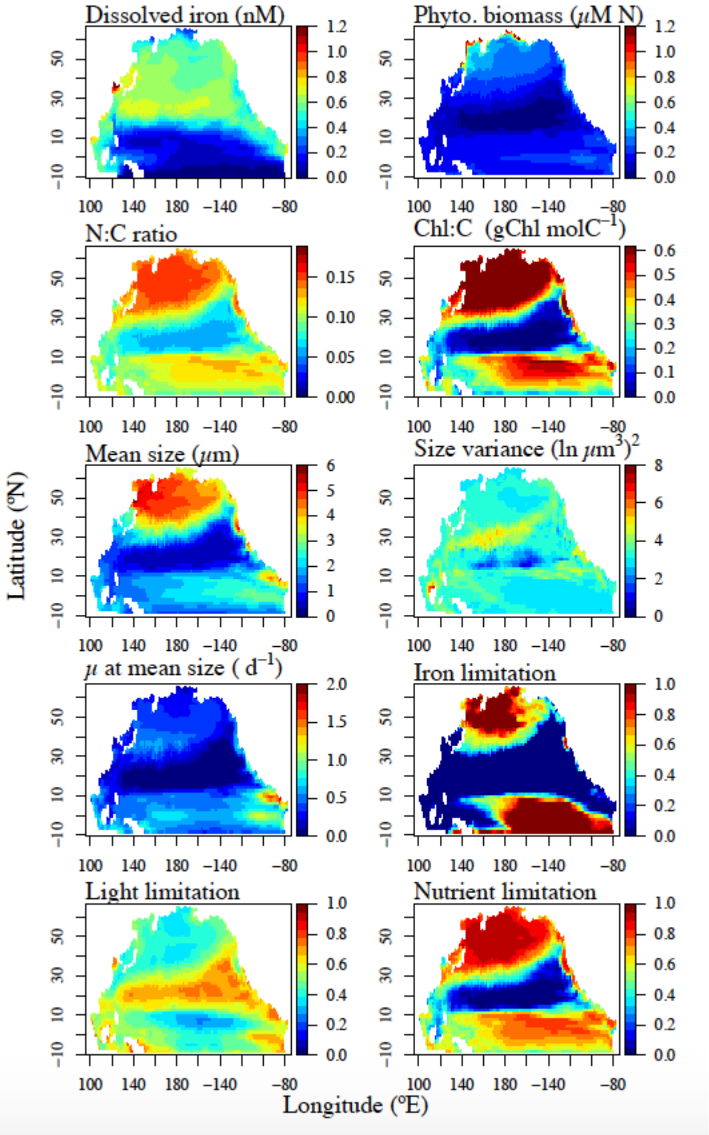

Fig. S4 Annual spatial patterns of dissolved iron, phytoplankton biomass, nitrogen-to-carbon ratios, chlorophyll-to-carbon ratios, phytoplankton mean ESD, the variance of log cell volume, growth rate at mean size, whether iron is limiting (1 indicating iron limitation and 0 indicating nitrogen limitation), and the extent of light and nutrient limitations (1 means no-limiting and 0 means 100% limiting and no growth). Note that we set that phytoplankton growth can be limited only by iron or nitrogen, whichever is more limiting. But the phytoplankton growth rate is a multiplicative function of light and nutrient terms, indicating both light and nutrient can be limiting.

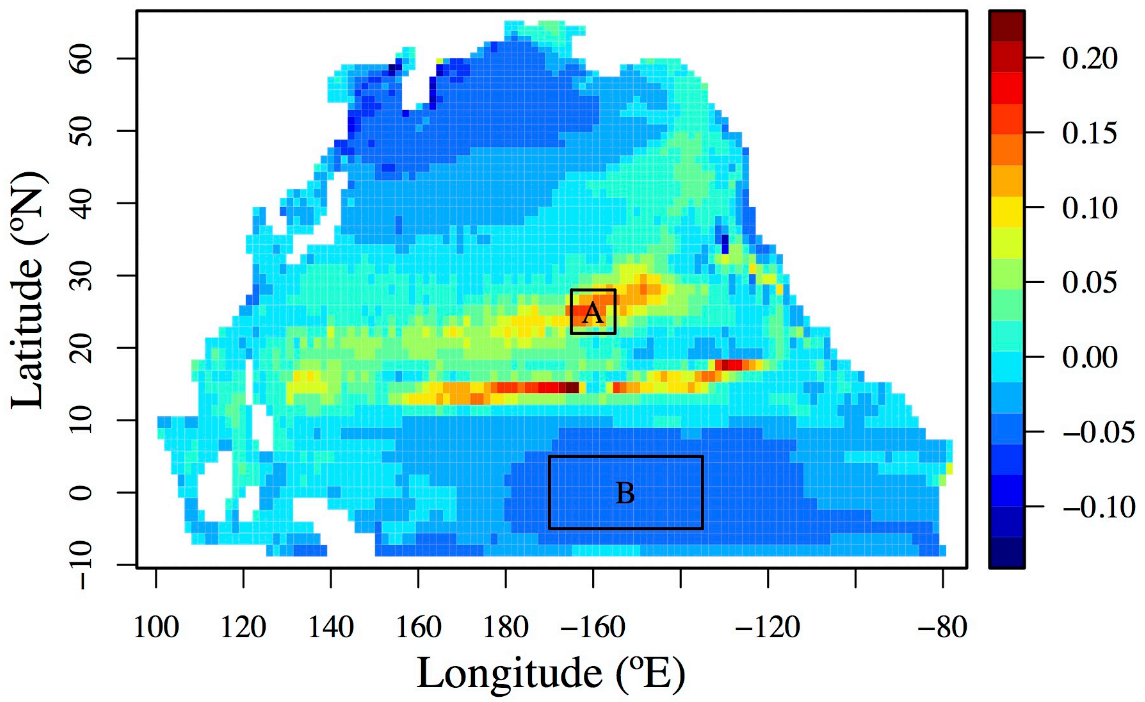

Fig. S5 Logarithmic ratios of NPP between the high- and low-diversity treatment (the same as Fig. 4d) overlaid with two hotspots.

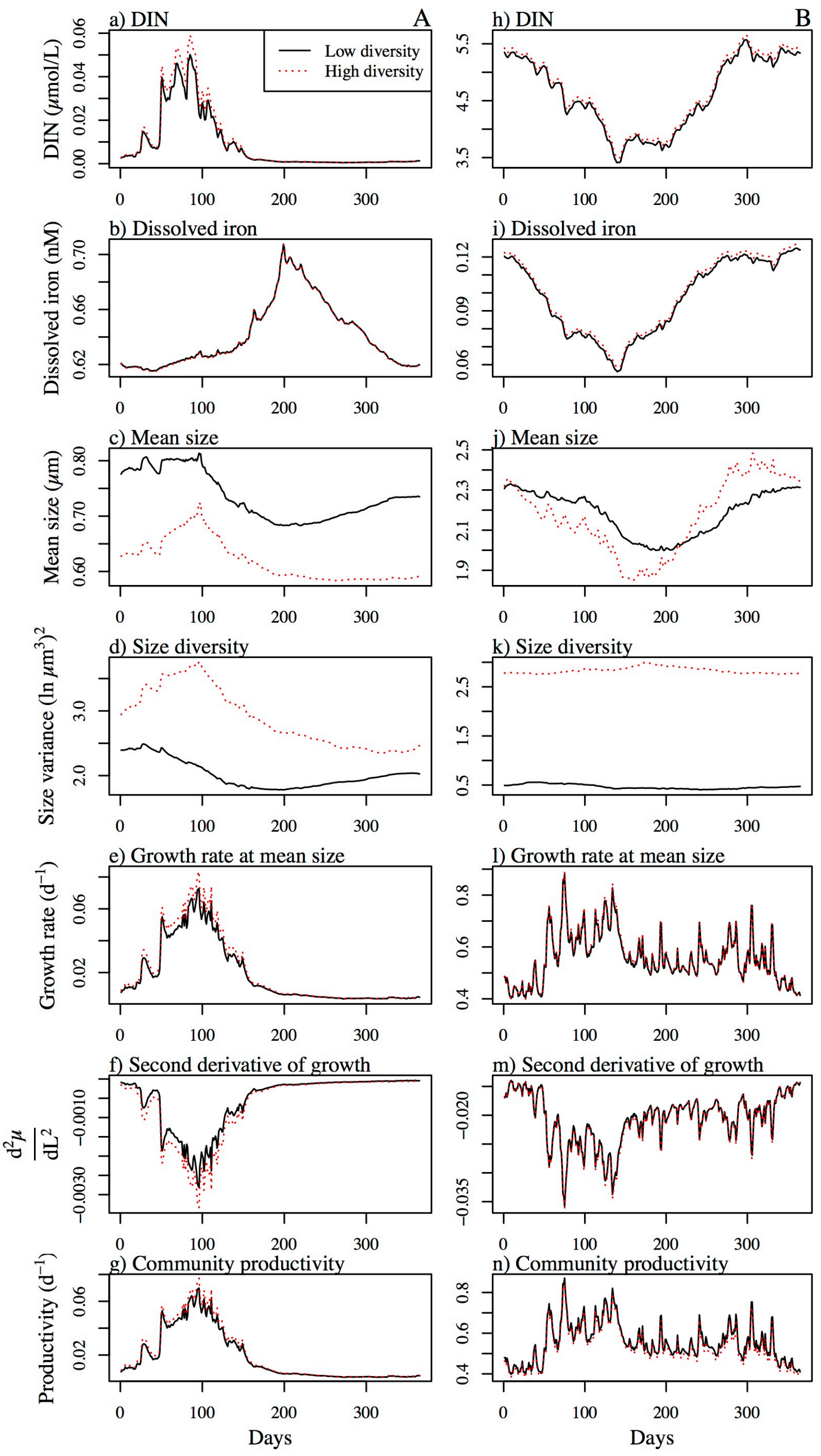

Fig. S6 Seasonal changes of dissolved inorganic nitrogen (DIN), dissolved iron, mean size, size diversity, growth rate at mean size, second derivative of growth rate at mean size, and community productivity at two selected hotspots where negative and positive effects of diversity are most evident (*see* Fig. S5).

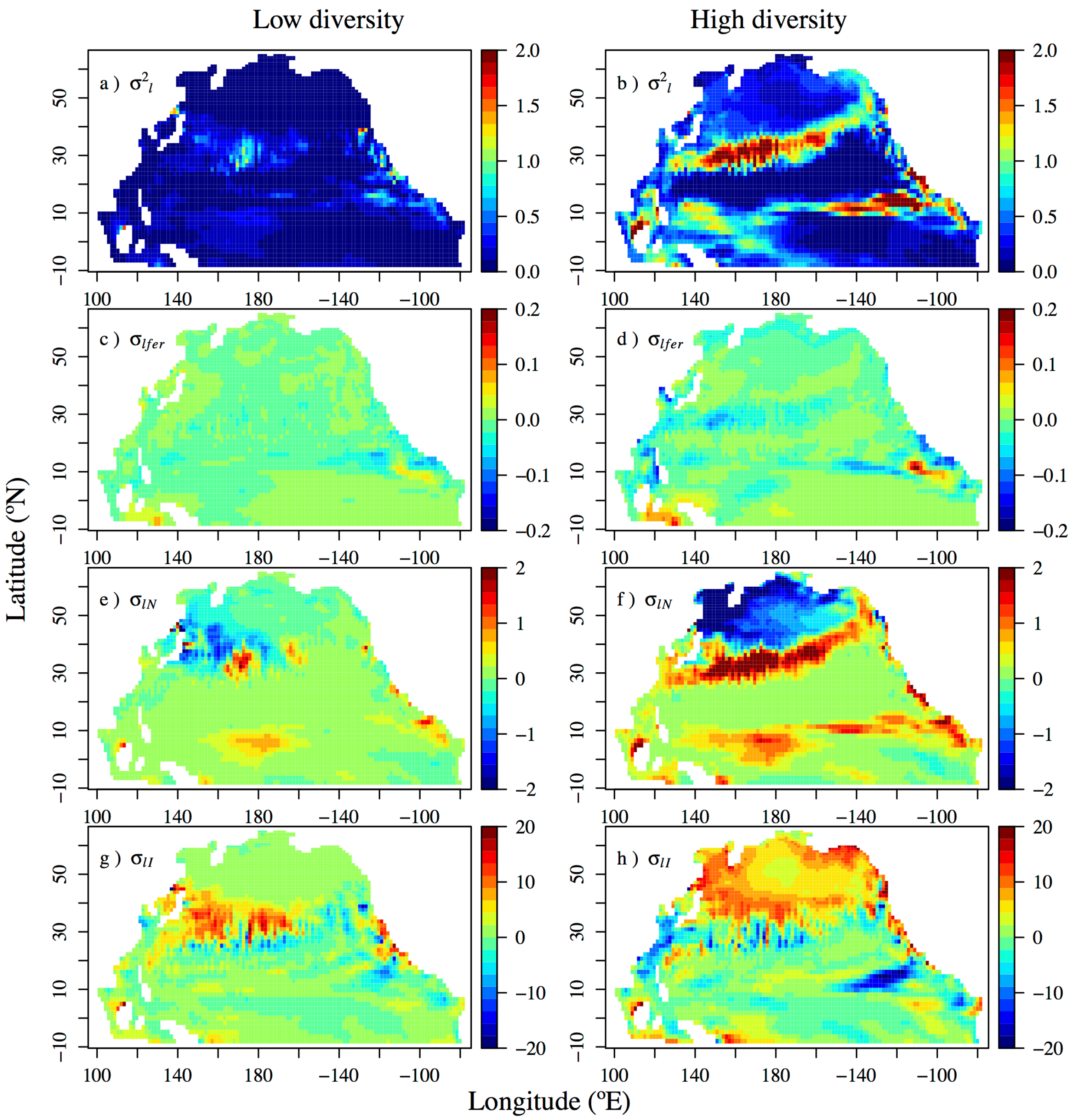

Fig. S7 Spatial comparison of temporal (a, b) variances of mean size and covariances (c, d) between mean size and light, (e, f) between mean size and nitrate, and (g, h) between mean size and iron within the surface mixed layer in the high- and low-diversity treatments.

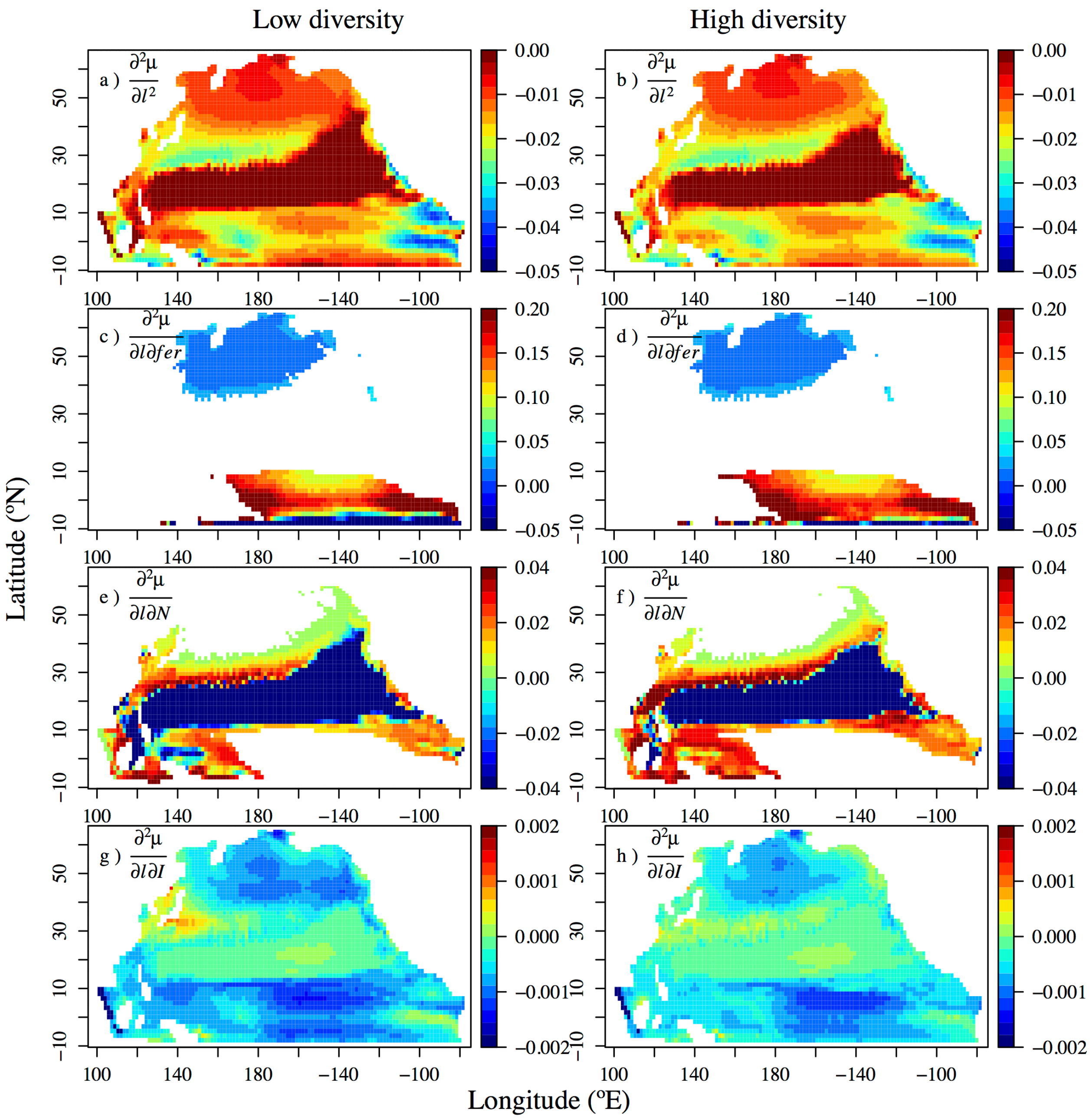

Fig. S8 Spatial comparisons of (a, b) $\left.\frac{\partial^2\mu}{\partial \bar{l}^2}\right|_{\bar{l}=\bar{l}_0}$, (c, d) $\left.\frac{\partial^2\mu}{\partial \bar{l}\partial fer}\right|_{\bar{l}=\bar{l}_0, fer=fer_0}$, (e, f) $\left.\frac{\partial^2\mu}{\partial \bar{l}\partial N}\right|_{\bar{l}=\bar{l}_0, N=N_0}$, (g, h) $\left.\frac{\partial^2\mu}{\partial \bar{l}\partial I}\right|_{\bar{l}=\bar{l}_0, I=I_0}$ within the surface mixed layer in the high- and low-diversity treatments. X_0 represents the annual mean value of variable X.

S.4 Supporting references

Aumont, O. & Bopp, L. (2006). Globalizing results from ocean in situ iron fertilization studies. *Global Biogeochem. Cycles*, 20, GB2017, doi:10.1029/2005GB002591.

Behrenfeld, M.J. & Falkowski, P.G. (1997). Photosynthetic rates derived from satellite-based chlorophyll concentration. *Limnol. Oceanogr.*, 42, 1–20.

Boyer, T.P., Antonov, J. I., Baranova, O. K., Coleman, C., Garcia, H. E., Grodsky, A., Johnson, D. R., Locarnini, R. A., Mishonov, A. V., O'Brien, T.D., Paver, C.R., Reagan, J.R., Seidov, D., Smolyar, I. V. & Zweng, M. M. (2013). World Ocean Database 2013, NOAA Atlas NESDIS 72, S. Levitus, Ed., A. Mishonov, Technical Ed.; Silver Spring, MD, 209 pp., http://doi.org/10.7289/V5NZ85MT

Buitenhuis, E.T., Rivkin, R.B., Sailley, S., & C. Le Quere. (2010). Biogeochemical fluxes through microzooplankton. *Global Biogeochem. Cycles*, 24, GB4015, doi:10.1029/2009GB003601.

Chai, F., Dugdale, R. C., Peng, T. H., Wilkerson, F. P., & Barber, R. T. (2002). One-dimensional ecosystem model of the equatorial Pacific upwelling system, Part I: model development and silicon and nitrogen cycle. *Deep-Sea Res.* II, 49, 2713–2745.

Chen, B. & Laws, E. A. (2017). Is there a difference of temperature sensitivity between marine phytoplankton and heterotrophs? *Limnol. Oceanogr.*, 62, 806–817, doi:10.1002/lno.10462.

Edwards, K. F., Thomas, M. K., Klausmeier, C.A. & Litchman, E. (2015). Light and growth in marine phytoplankton: allometric, taxonomic, and environmental variation. *Limnol. Oceanogr.*, 60, 540–552.

Fennel, K., Wilkin, J., Levin, J., Moisan, J., O'Reilly, J., & Haidvogel, D. (2006). Nitrogen cycling in the Middle Atlantic Bight: Results from a three-dimensional model and implications for the North Atlantic nitrogen budget. *Global Biogeochem. Cycles*, 20, GB3007, doi: 10.1029/2005GB002456.

Franks, P.J. (2015). Has Sverdrup's critical depth hypothesis been tested? Mixed layers vs. turbulent layers. *ICES J. Mar. Sci.*, 72, 1897–1907.

Flynn, K. J. (2003). Do we need complex mechanistic photoacclimation models for phytoplankton? *Limnol. Oceanogr.*, 48, 2243–2249.

Garcia, H.E., Locarnini, R. A., Boyer, T. P., Antonov, J. I., Baranova, O.K., Zweng, M.M., Reagan, J.R., Johnson, D.R. (2014). World Ocean Atlas 2013, Volume 4: Dissolved Inorganic Nutrients (phosphate, nitrate, silicate). S. Levitus, Ed., A. Mishonov Technical Ed.; *NOAA Atlas NESDIS* 76, 25 pp.

Gregg, W.W. (2008). Assimilation of SeaWiFS ocean chlorophyll data into a three-dimensional global ocean model. *J. Mar. Syst.*, 69, 205–225.

Huston, M.: A general hypothesis of species diversity, Am. Nat., 113, 81–101, 1979.

Large, W.G. & Yeager, S.G. (2009). The global climatology of an interannually varying air–sea flux data set. Clim. Dyn., 33, 341–364.

Luo, C., Mahowald, N., Bond, T., Chuang, P. Y., Artaxo, P., Siefert, R., Chen, Y., & Schauer, J. (2008). Combustion iron distribution and deposition. *Global Biogeochem. Cycles*, 22, GB1012, doi:10.1029/2007GB002964.

Marañón, E., Cermeño, P., López-Sandoval, D. C., Rodríguez- Ramos, T., Sobrino, C., Huete-Ortega, M., Blanco, J. M., & Rodríguez, J. (2013). Unimodal size scaling of phytoplankton growth and the size dependence of nutrient uptake and use. Ecol. Lett., 16, 371–379.

Merico, A., Brandt, G., Smith, S. L., & Oliver, M. (2014). Sustaining diversity in trait-based models of phytoplankton communities. *Front. Ecol. Evol.*, 2, 59, doi: 10.3389/fevo.2014.00059.

Morel, A. & Berthon, J.F. (1989). Surface pigments, algal biomass profiles, and potential production of the euphotic layer: Relationships reinvestigated in view of remote-sensing applications. *Limnol. Oceanogr.*, 34,1545–1562.

Nickelsen, L., Keller, D. & Oschlies, A. (2015). A dynamic marine iron cycle module coupled to the University of Victoria Earth System Model: the Kiel Marine Biogeochemical Model 2 for UVic 2.9. *Geosci. Model Dev.*, 8, 1357–1381, doi:10.5194/gmd-8-1357-2015.

Norberg, J., Swaney, D.P., Dushoff, J., Lin, J., Casagrandi, R., & Levin, S. A. (2001). Phenotypic diversity and ecosystem functioning in changing environments: a theoretical framework. *Proc. Nat. Acad. Sci.*, 98, 11376–11381.

Pahlow, M., Dietze, H. & Oschlies, A. (2013). Optimality-based model of phytoplankton growth and diazotrophy. *Mar. Ecol. Prog. Ser.*, 489, 1–16.

Smith, S. L., Vallina, S. M., & Merico, A. (2016). Phytoplankton size-diversity mediates an emergent trade-off in ecosystem functioning for rare versus frequent disturbances. *Sci. Rep.*, 6, 34170, doi: 10.1038/srep34170.

Vallina, S.M., Cermeno, P., Dutkiewicz, S., Loreau, M. & Montoya, J.M. (2017). Phytoplankton functional diversity increases ecosystem productivity and stability. *Ecol. Mod.*, 361, 184–196.

Ward, B.A. (2015). Temperature-correlated changes in phytoplankton community structure are restricted to polar waters. *PloS one*, 10, p.e0135581.

Ward, B.A., Dutkiewicz, S., Jahn, O. & Follows, M.J. (2012). A size-structured food-web model for the global ocean. *Limnol. Oceanogr.*, 57, 1877-1891.

Westberry, T., Behrenfeld, M.J., Siegel, D.A. & Boss, E. (2008). Carbon-based primary productivity *modeling* with vertically resolved photoacclimation. *Global Biogeochem. Cycles*, 22, GB2024, doi:10.1029/2007GB003078.

Wirtz K.W. (2014) A biomechanical and optimality-based derivation of prey-size dependencies in planktonic prey selection and ingestion rates. *Mar Ecol Prog Ser*, 507, 81-94.

Yachi, S. & Loreau, M. (1999). Biodiversity and ecosystem productivity in a fluctuating environment: the insurance hypothesis. *Proc. Nat. Acad. Sci.*, 96, 1463–1468.
